# Supplementary material for: A redox-neutral catechol synthesis
Source: Nat Commun. 2017 Jan 27;8:14227. doi: 10.1038/ncomms14227 (PMC5290158; doi:10.1038/ncomms14227)
Supplement: Supplementary Information — Supplementary figures, supplementary tables, supplementary discussion, supplementary methods and supplementary references. [file ncomms14227-s1.pdf]

Supplementary Figures

1q

N-methyl-*N*-phenoxyacetamide

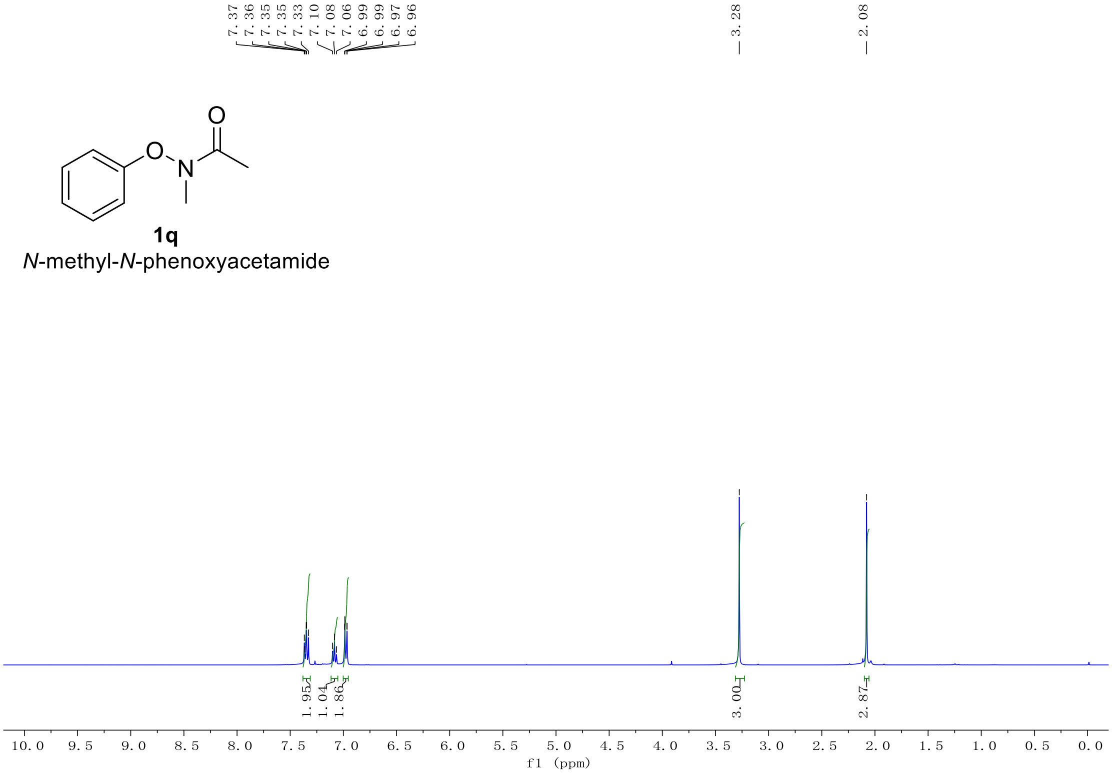

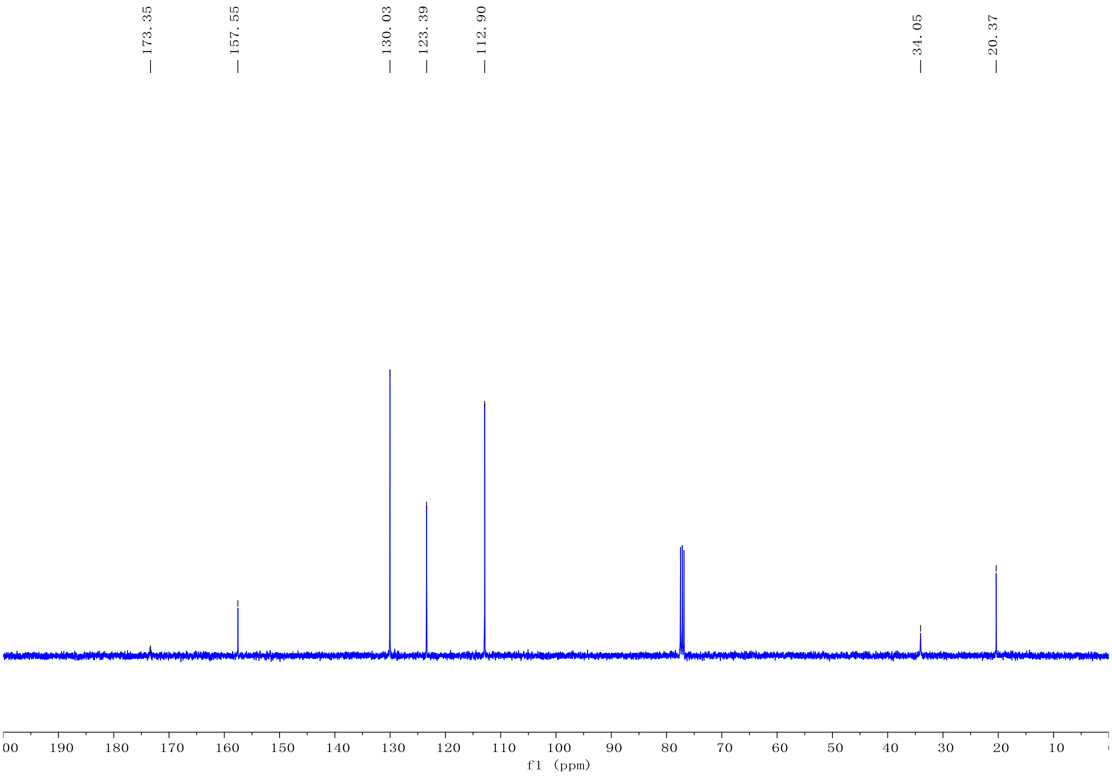

Supplementary Figure 1. ^{1}H and ^{13}C NMR spectra for compound **1q**.

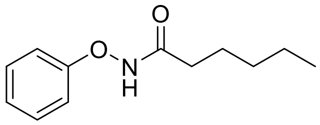

1r
N-phenoxyhexanamide

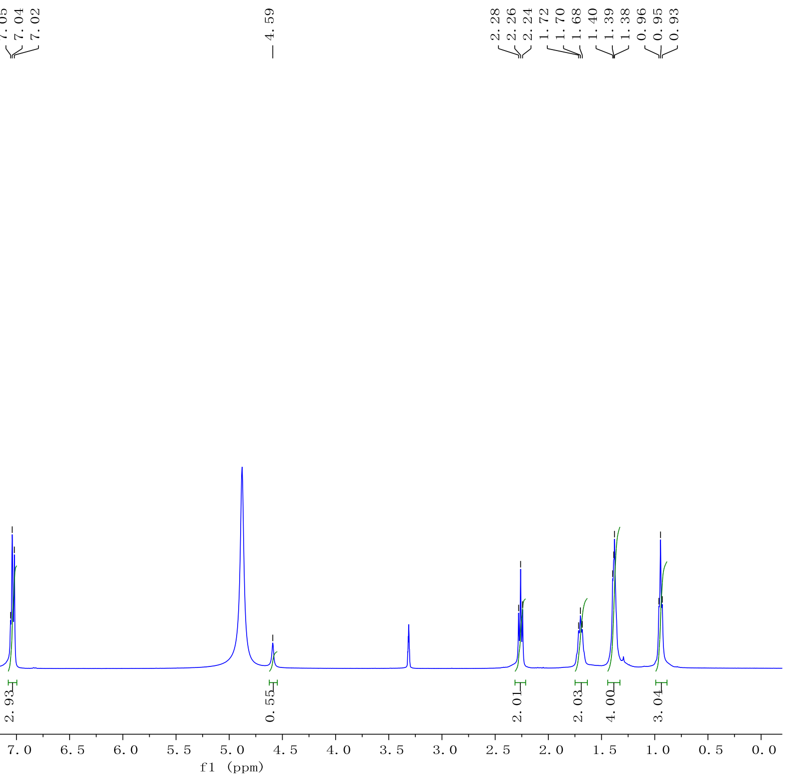

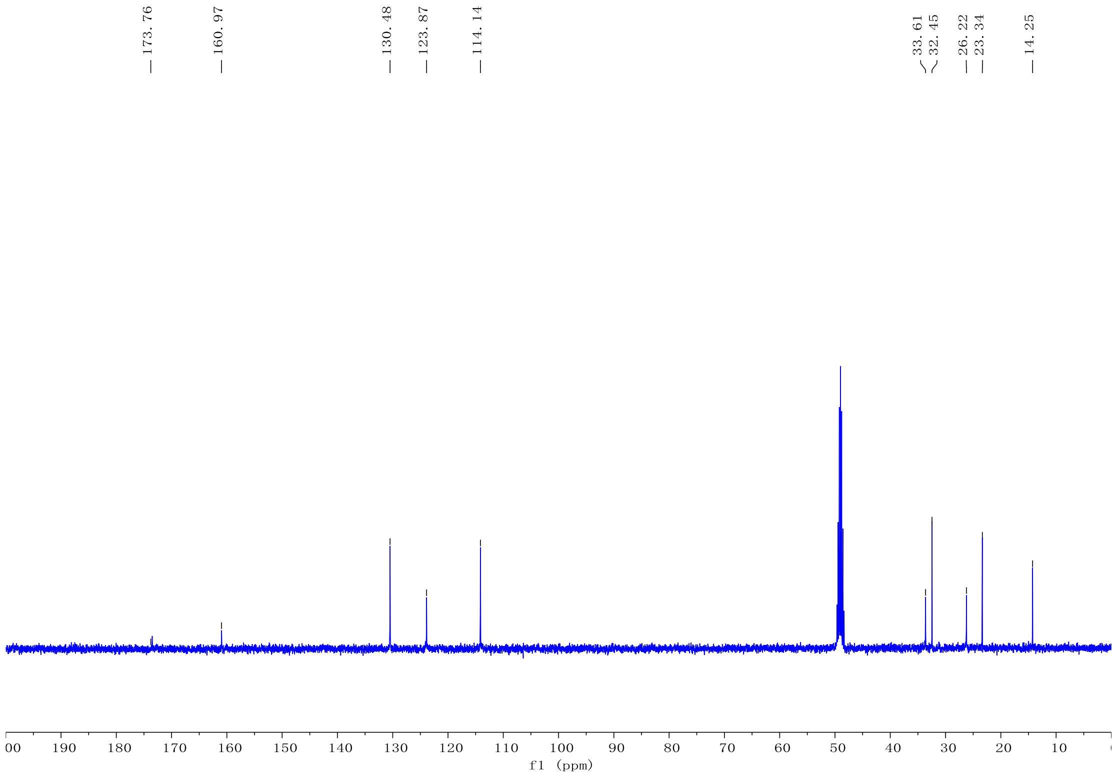

Supplementary Figure 2. ^{1}H and ^{13}C NMR spectra for compound **1r**.

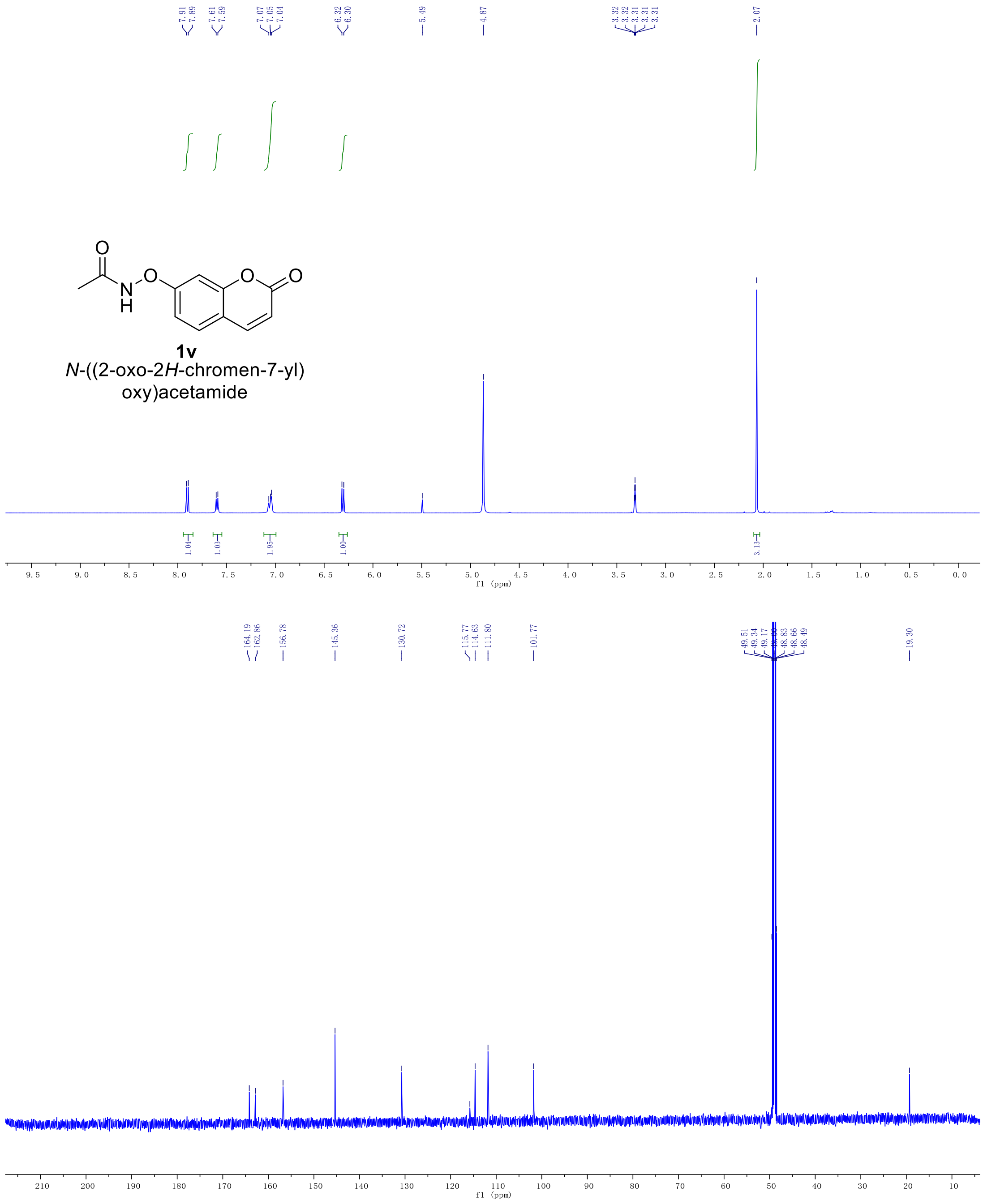

Supplementary Figure 3. ^{1}H and ^{13}C NMR spectra for compound **1v**.

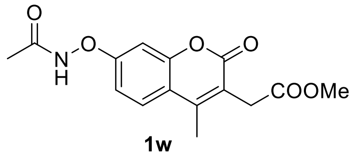

1w

methyl 2-(7-(acetamidooxy)-4-methyl-2-oxo-2*H*-chromen-3-yl)acetate

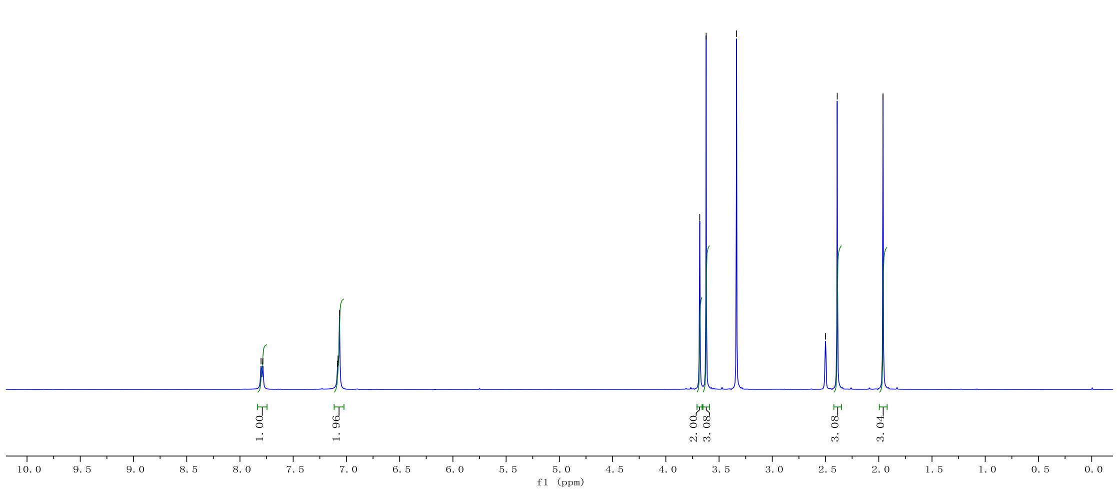

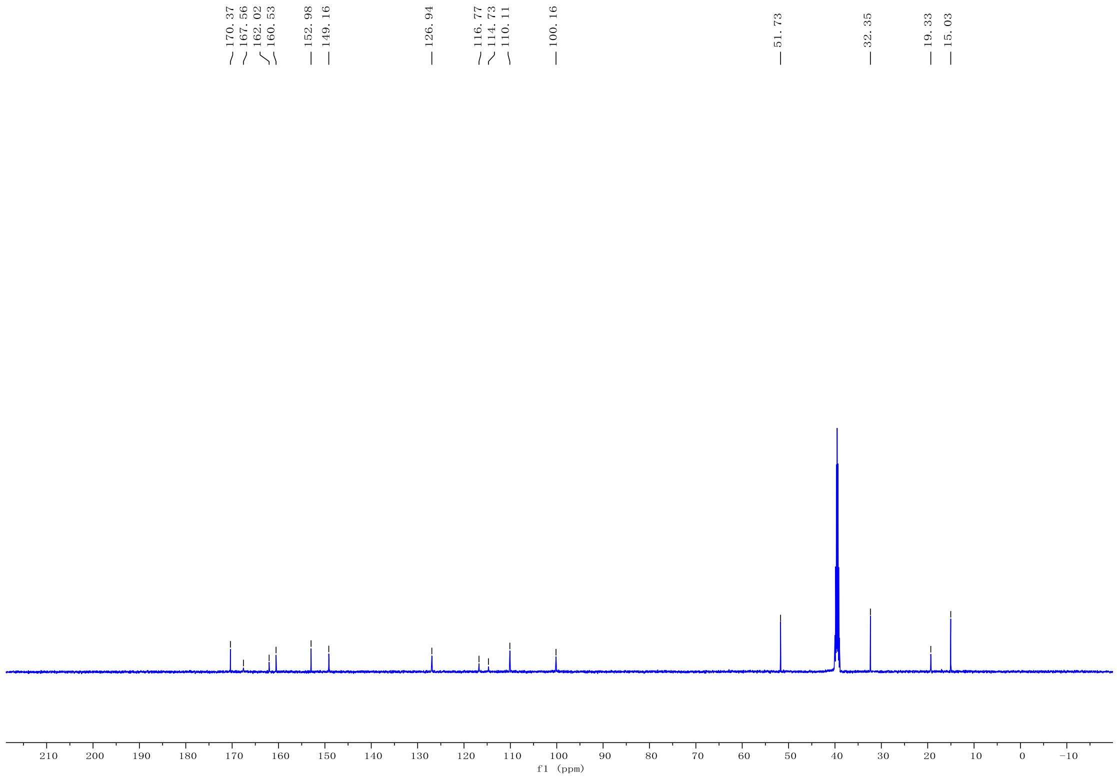

Supplementary Figure 4. ^{1}H and ^{13}C NMR spectra for compound **1w**.

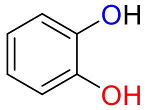

2a
pyrocatechol

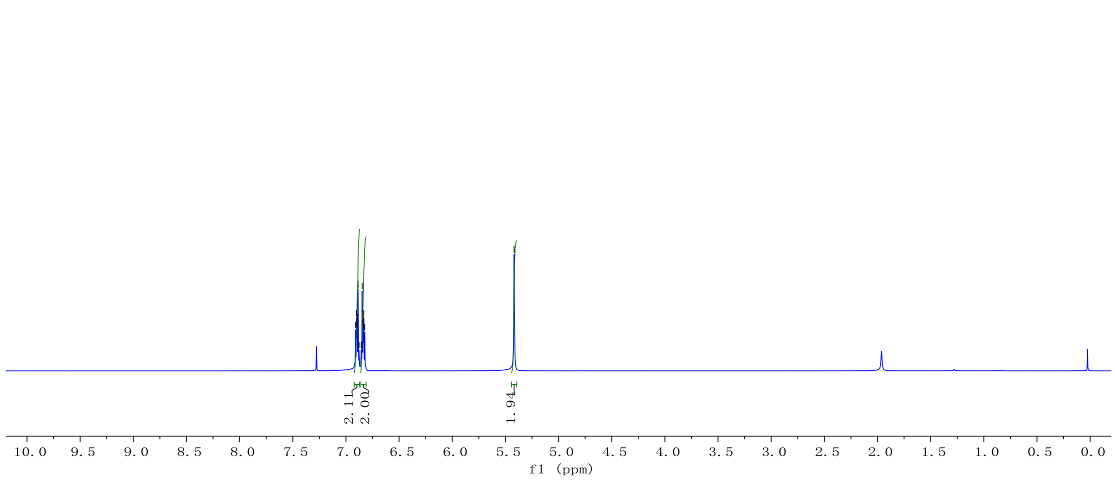

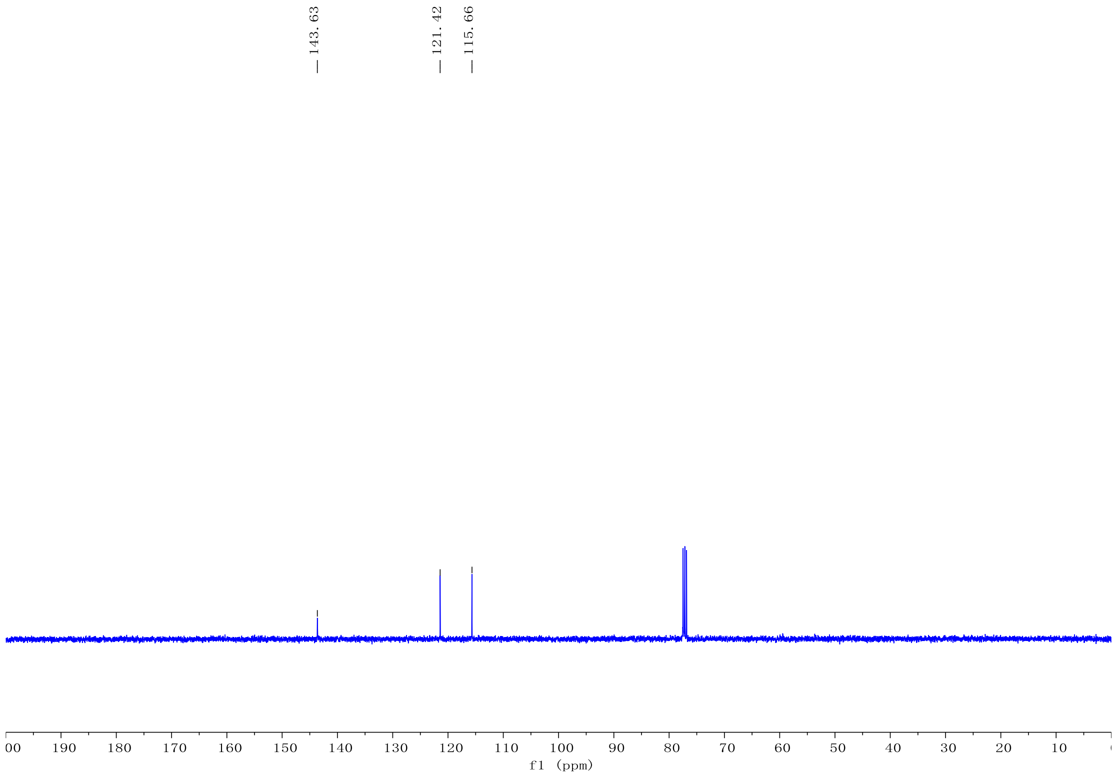

Supplementary Figure 5. ^{1}H and ^{13}C NMR spectra for compound **2a**.

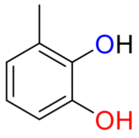

2b
3-methylbenzene-1,2-diol

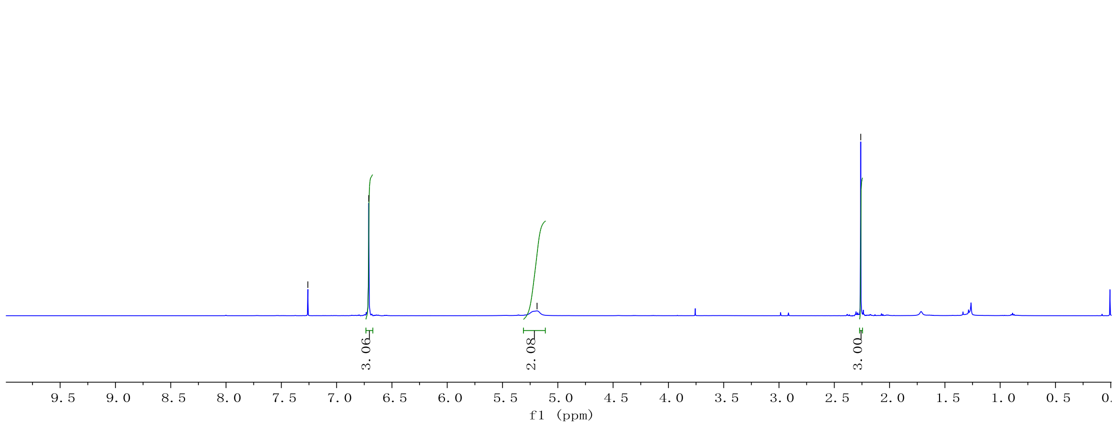

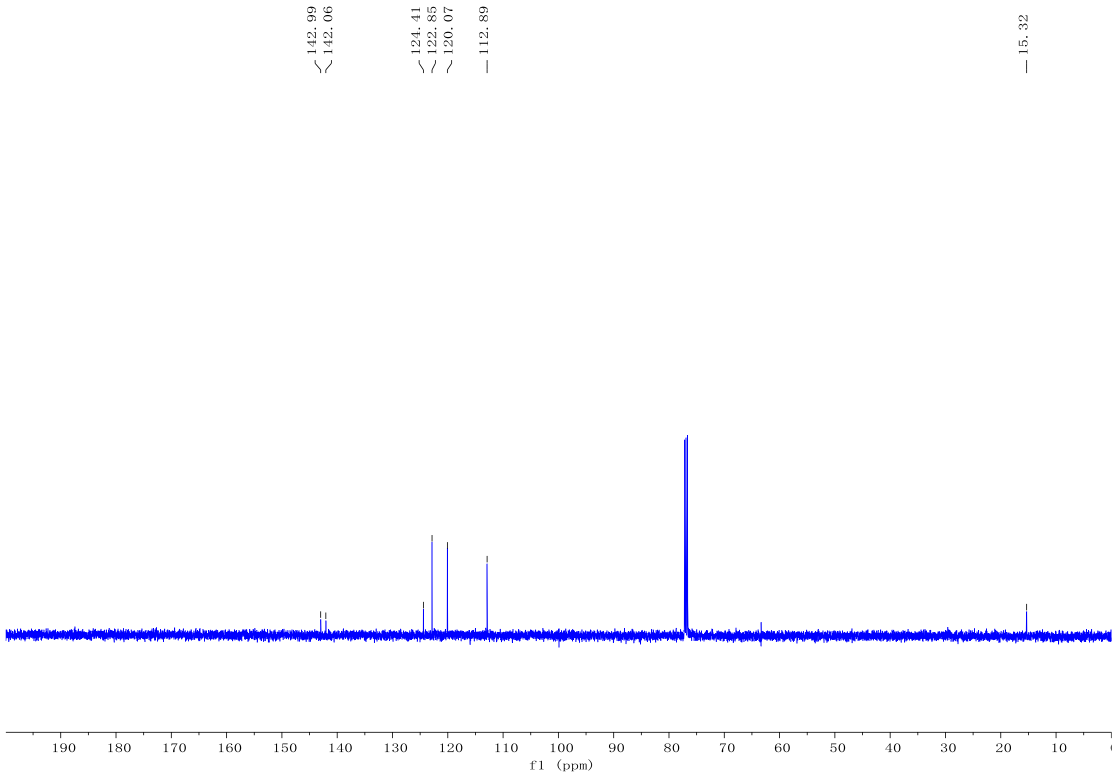

Supplementary Figure 6. ^{1}H and ^{13}C NMR spectra for compound **2b**.

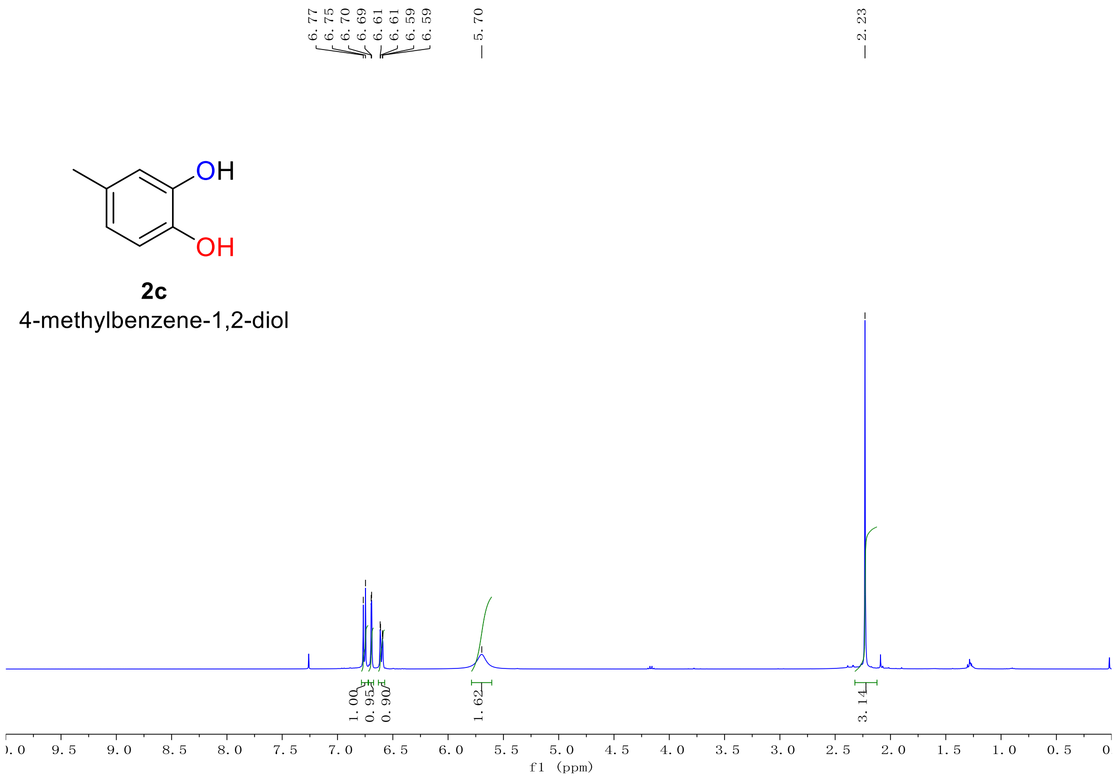

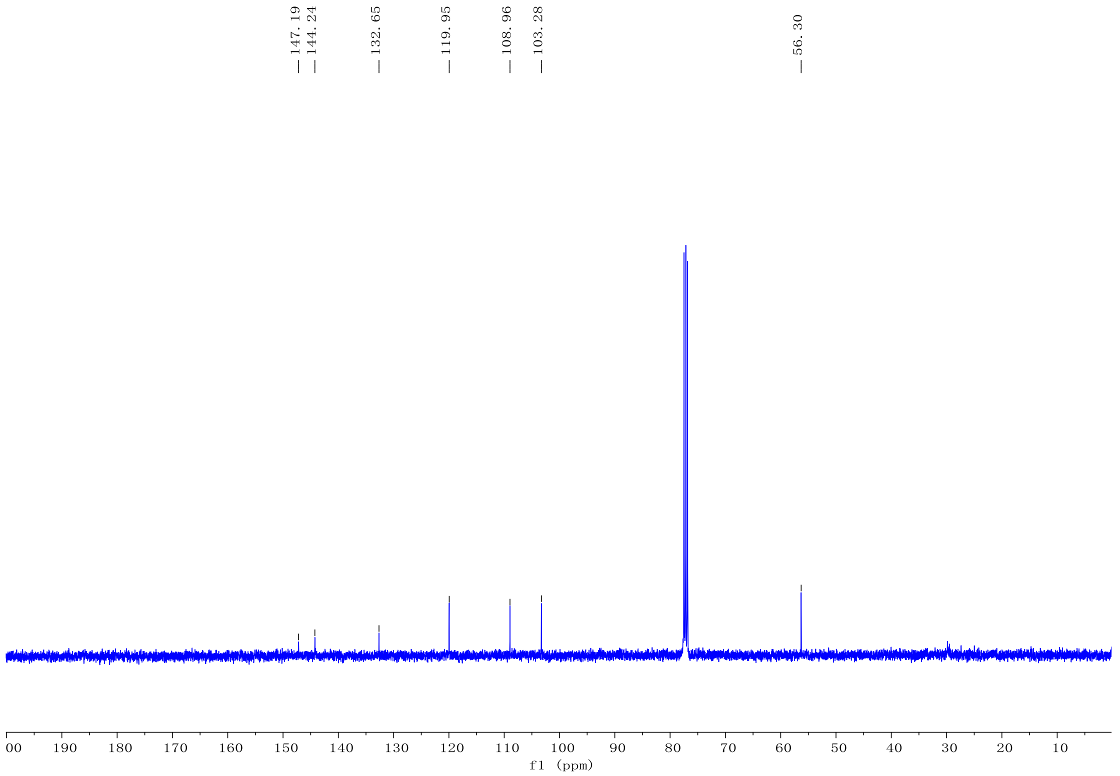

Supplementary Figure 7. ^{1}H and ^{13}C NMR spectra for compound **2c**.

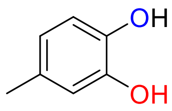

2d
4-methylbenzene-1,2-diol

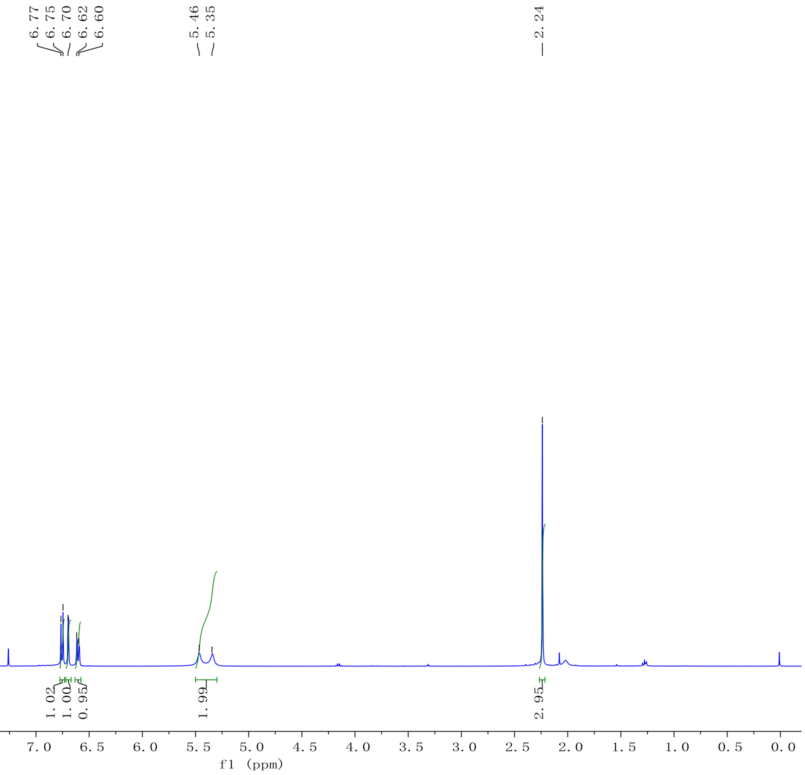

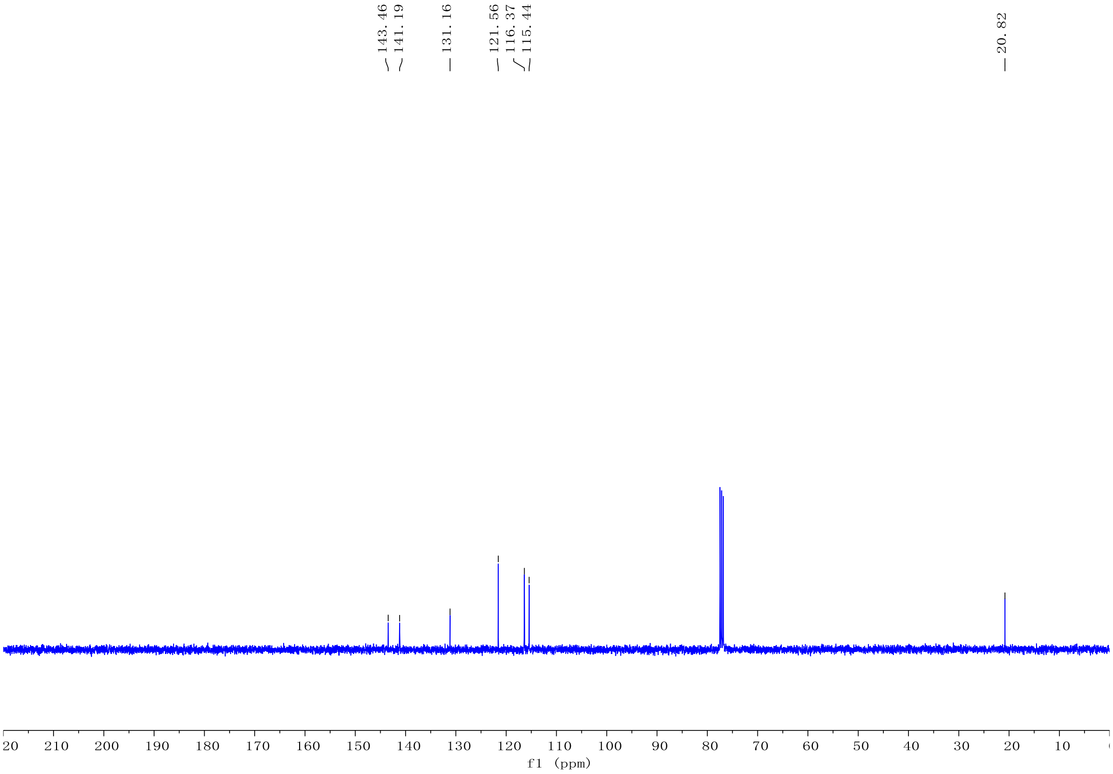

Supplementary Figure 8. ^{1}H and ^{13}C NMR spectra for compound **2d**.

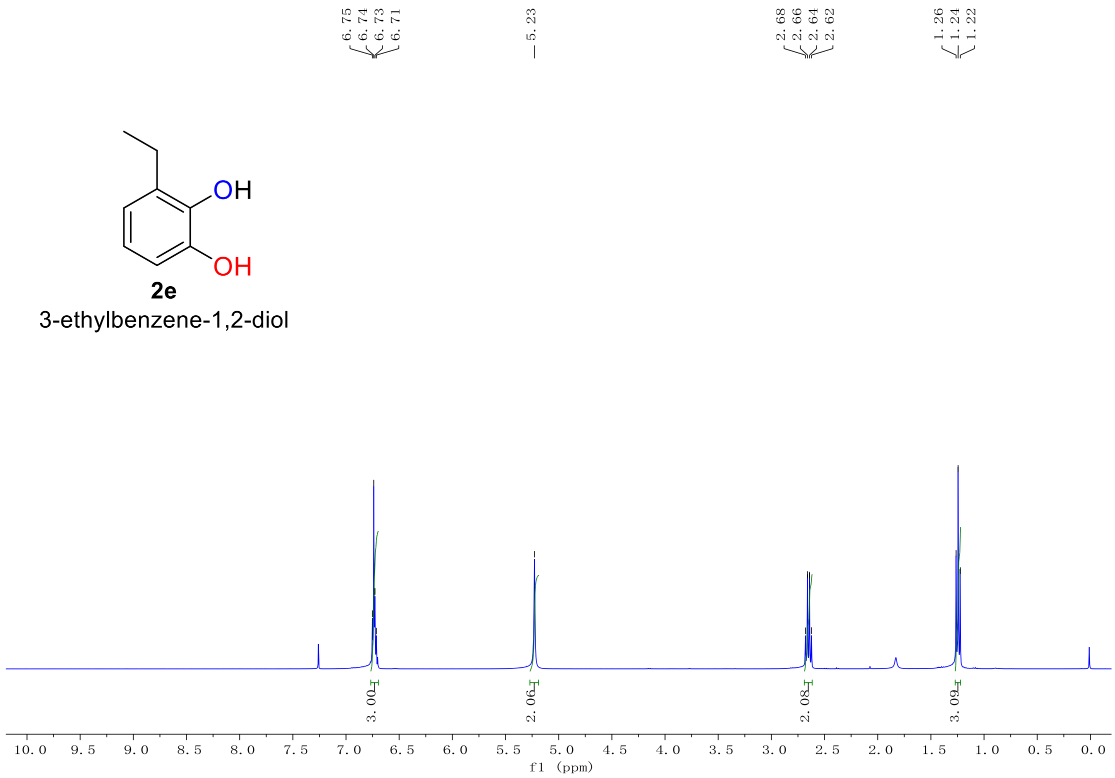

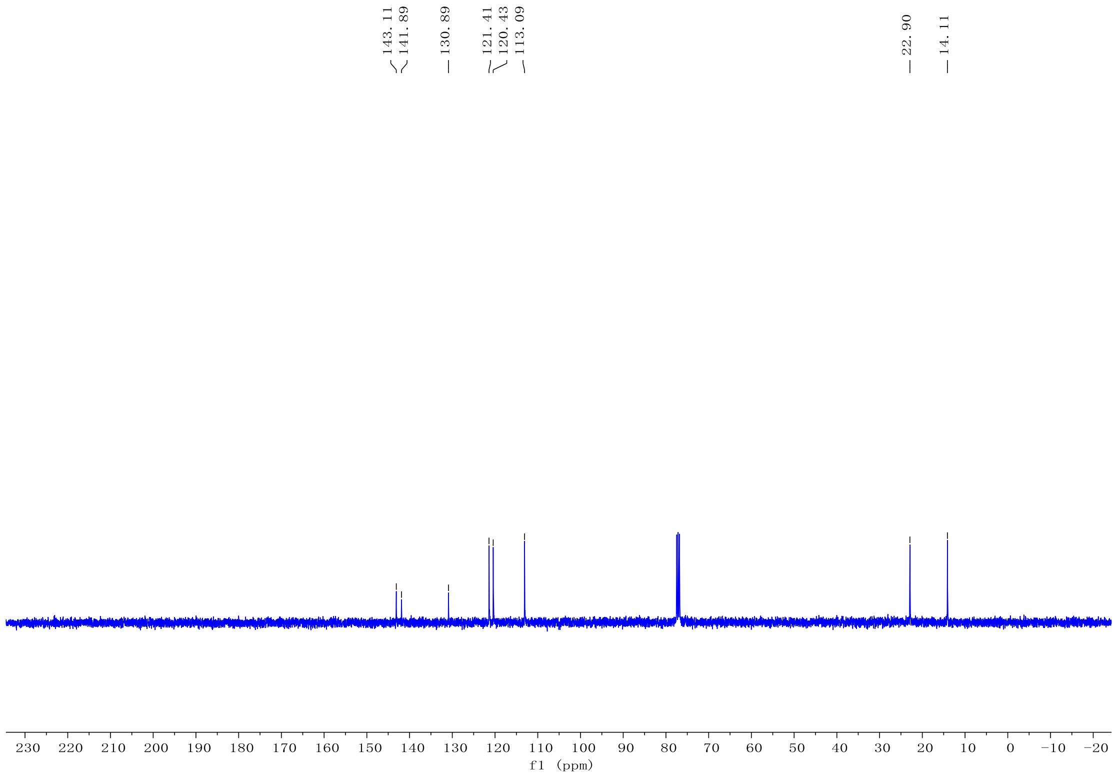

Supplementary Figure 9. ^{1}H and ^{13}C NMR spectra for compound **2e**.

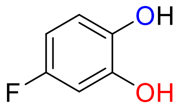

2f

4-fluorobenzene-1,2-diol

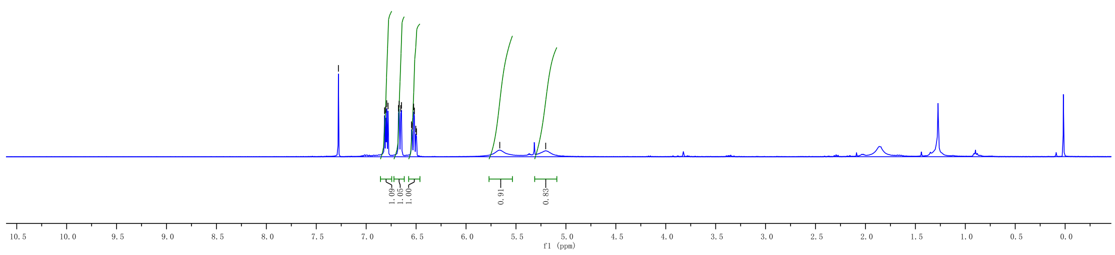

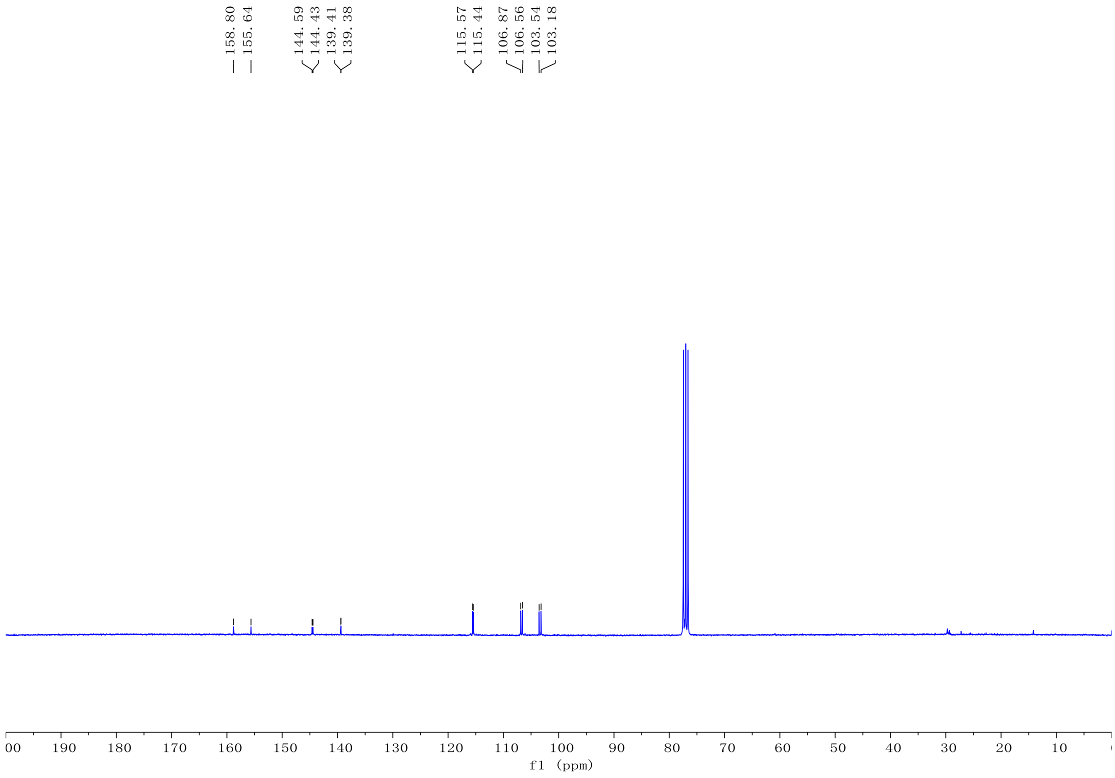

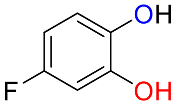

2f
4-fluorobenzene-1,2-diol

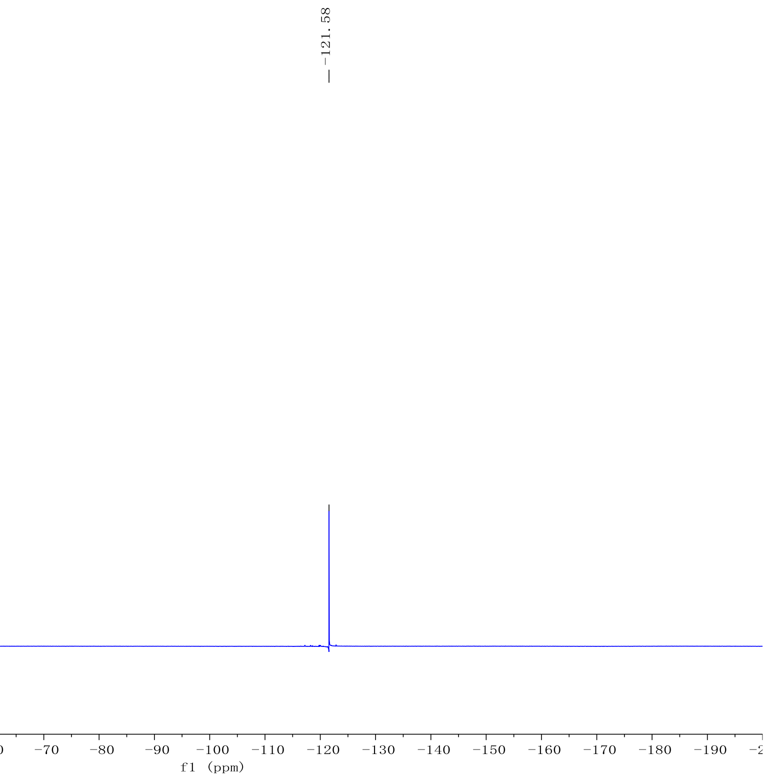

Supplementary Figure 10. ^{1}H , ^{13}C and ^{19}F NMR spectra for compound **2f**.

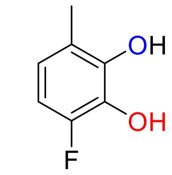

2g

3-fluoro-6-methylbenzene-1,2-diol

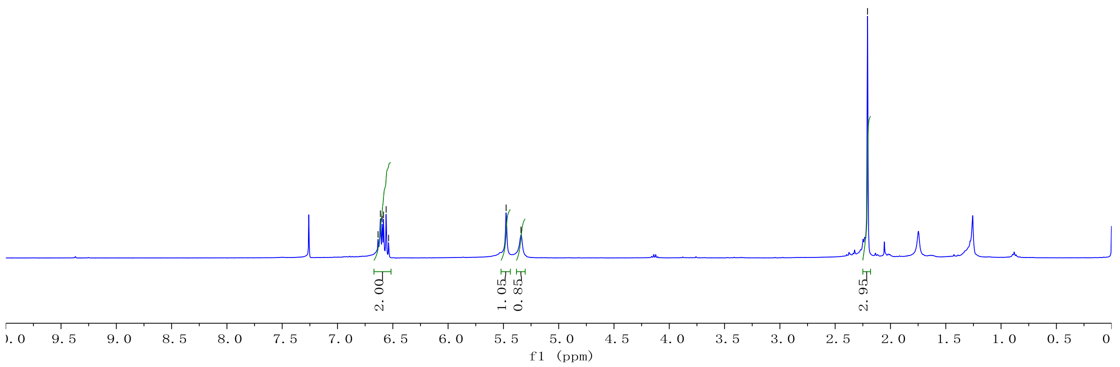

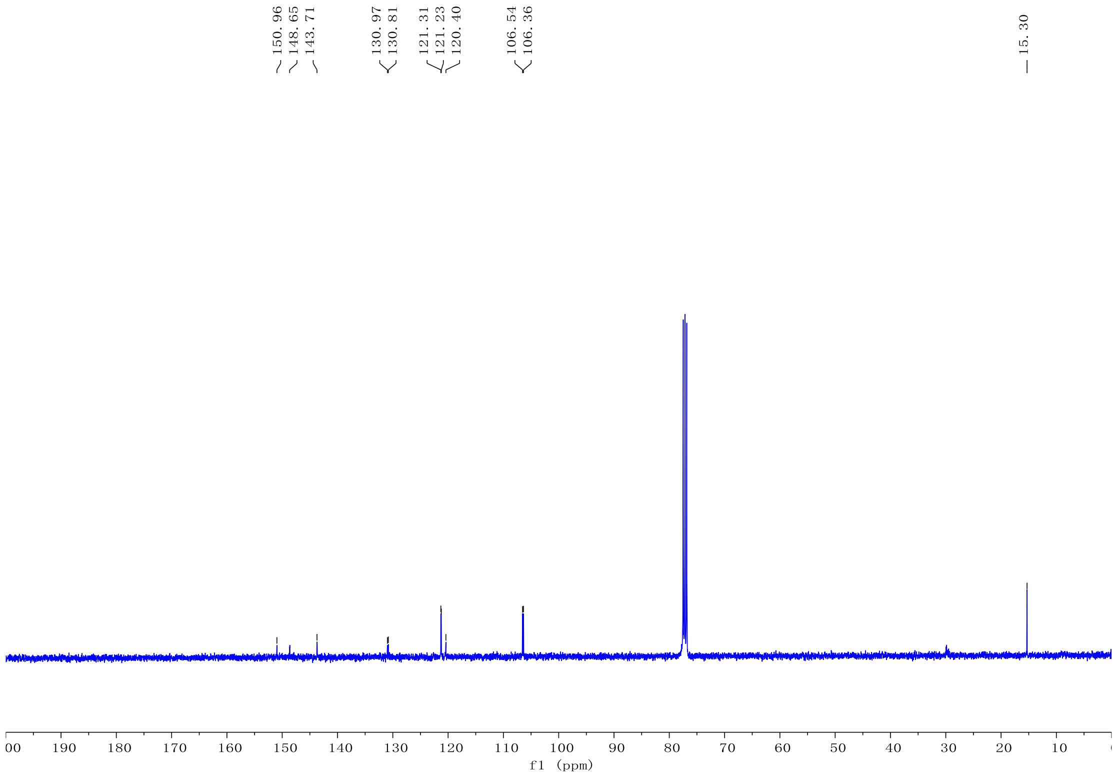

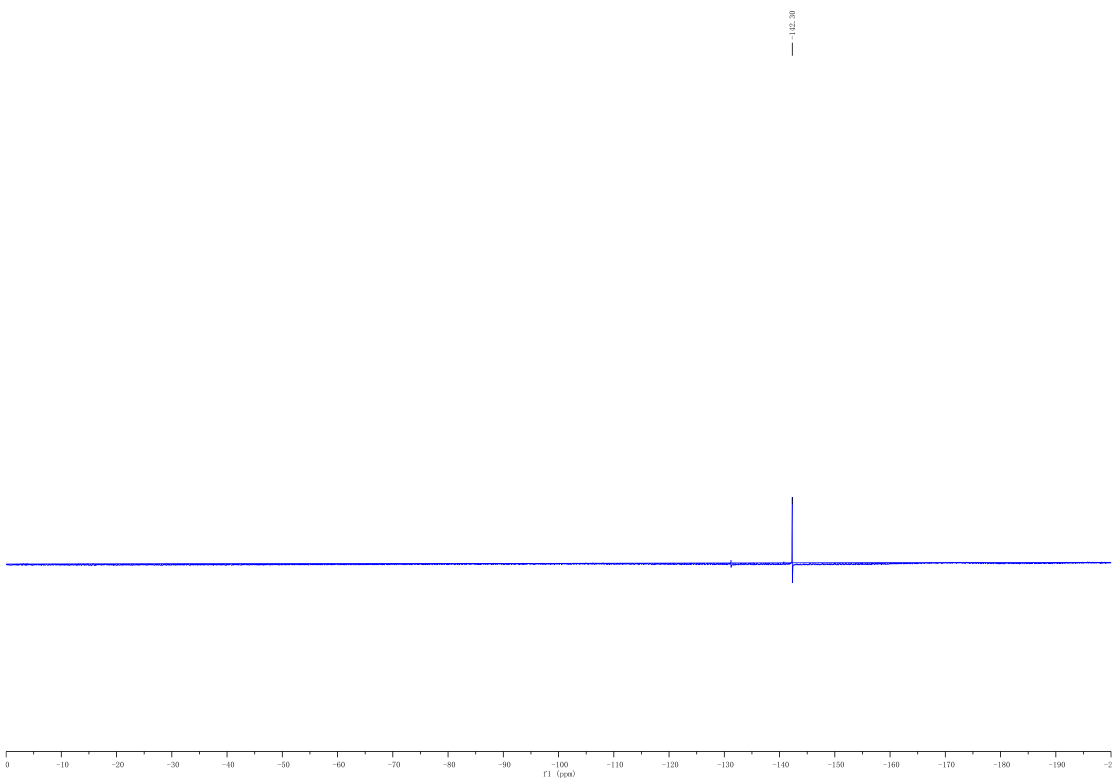

Supplementary Figure 11. ^{1}H , ^{13}C and ^{19}F NMR spectra for compound **2g**.

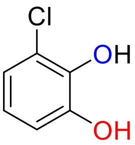

2h

3-chlorobenzene-1,2-diol

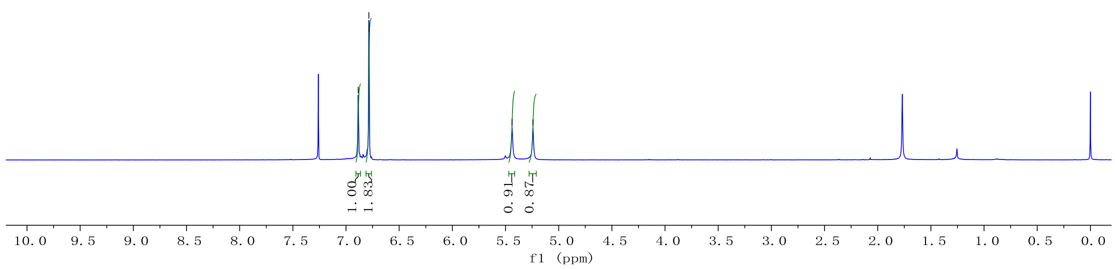

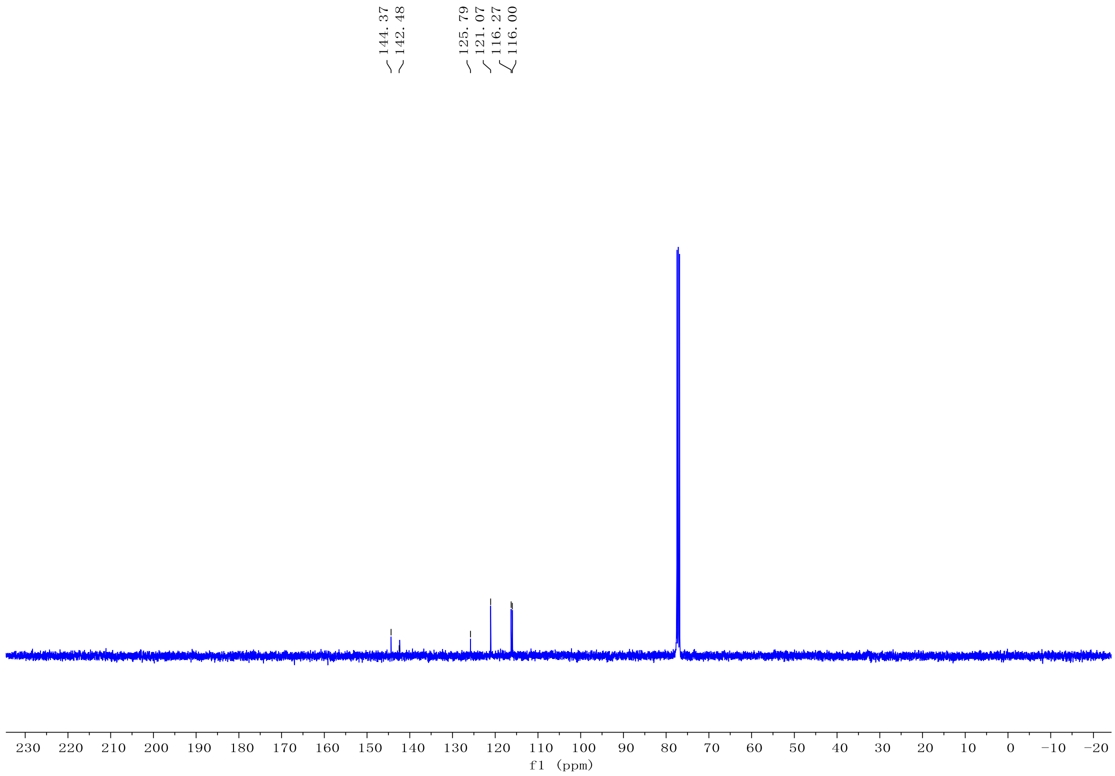

Supplementary Figure 12. ^{1}H , ^{13}C and ^{19}F NMR spectra for compound **2h**.

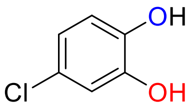

2i
4-chlorobenzene-1,2-diol

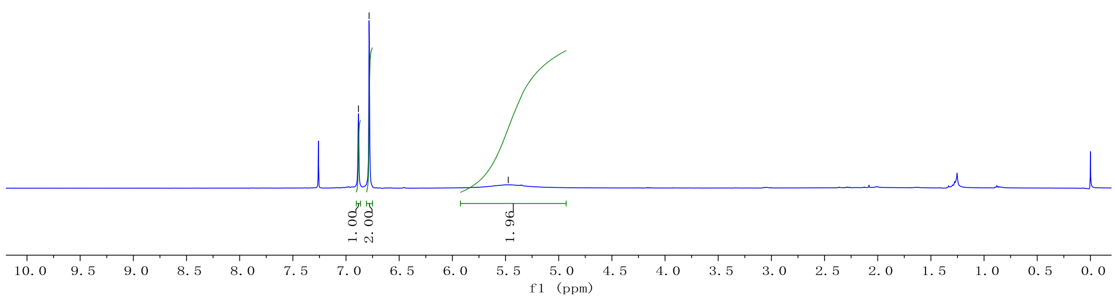

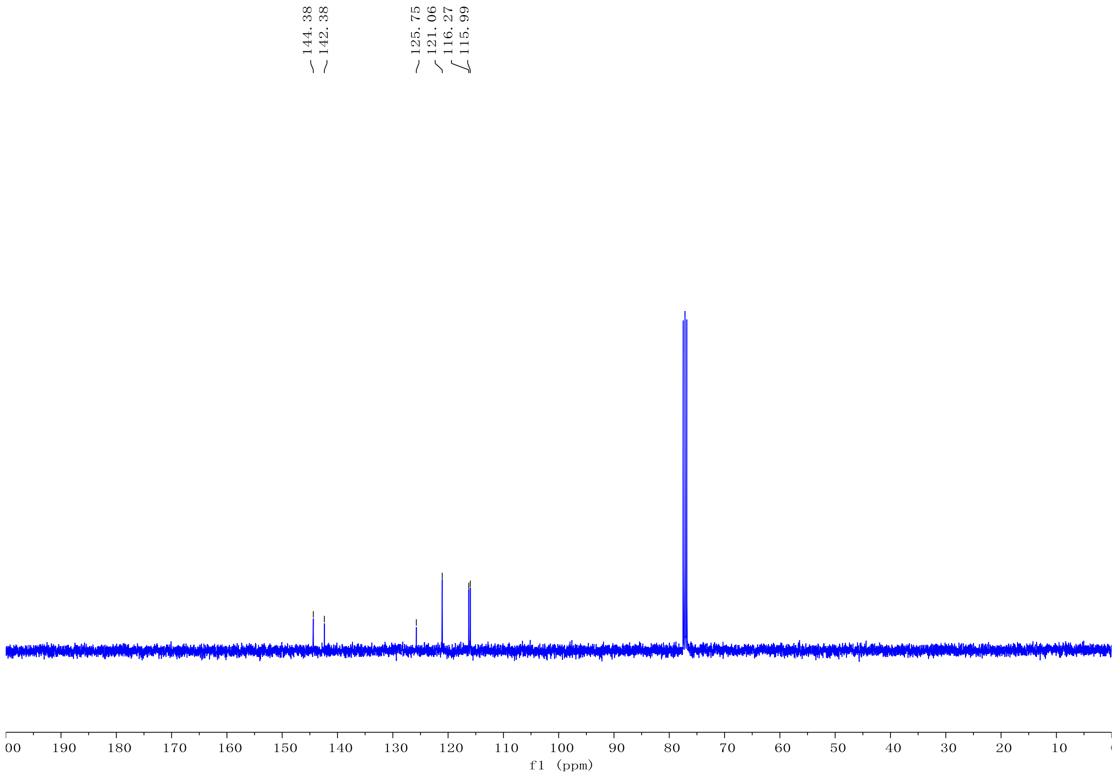

Supplementary Figure 13. ^{1}H and ^{13}C NMR spectra for compound **2i**.

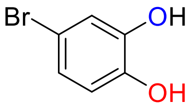

2j

4-bromobenzene-1,2-diol

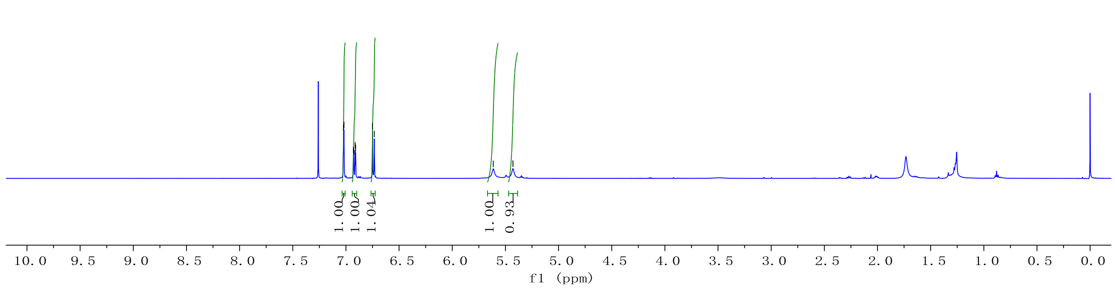

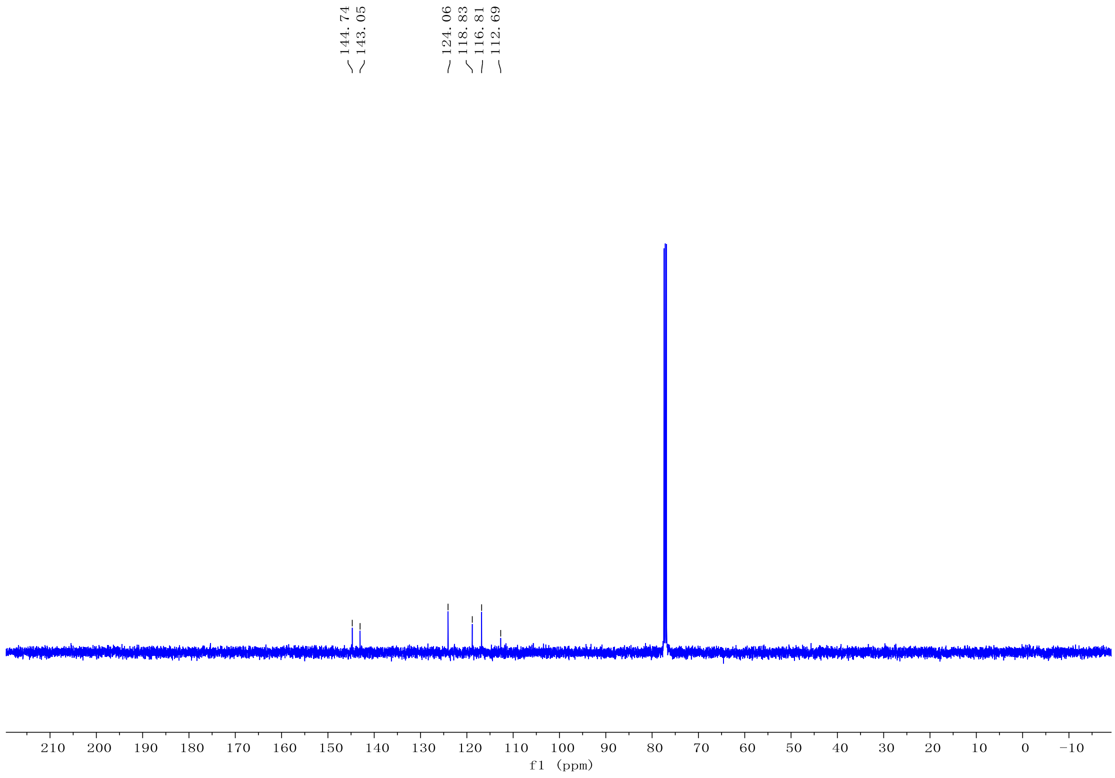

Supplementary Figure 14. ^{1}H and ^{13}C NMR spectra for compound **2j**.

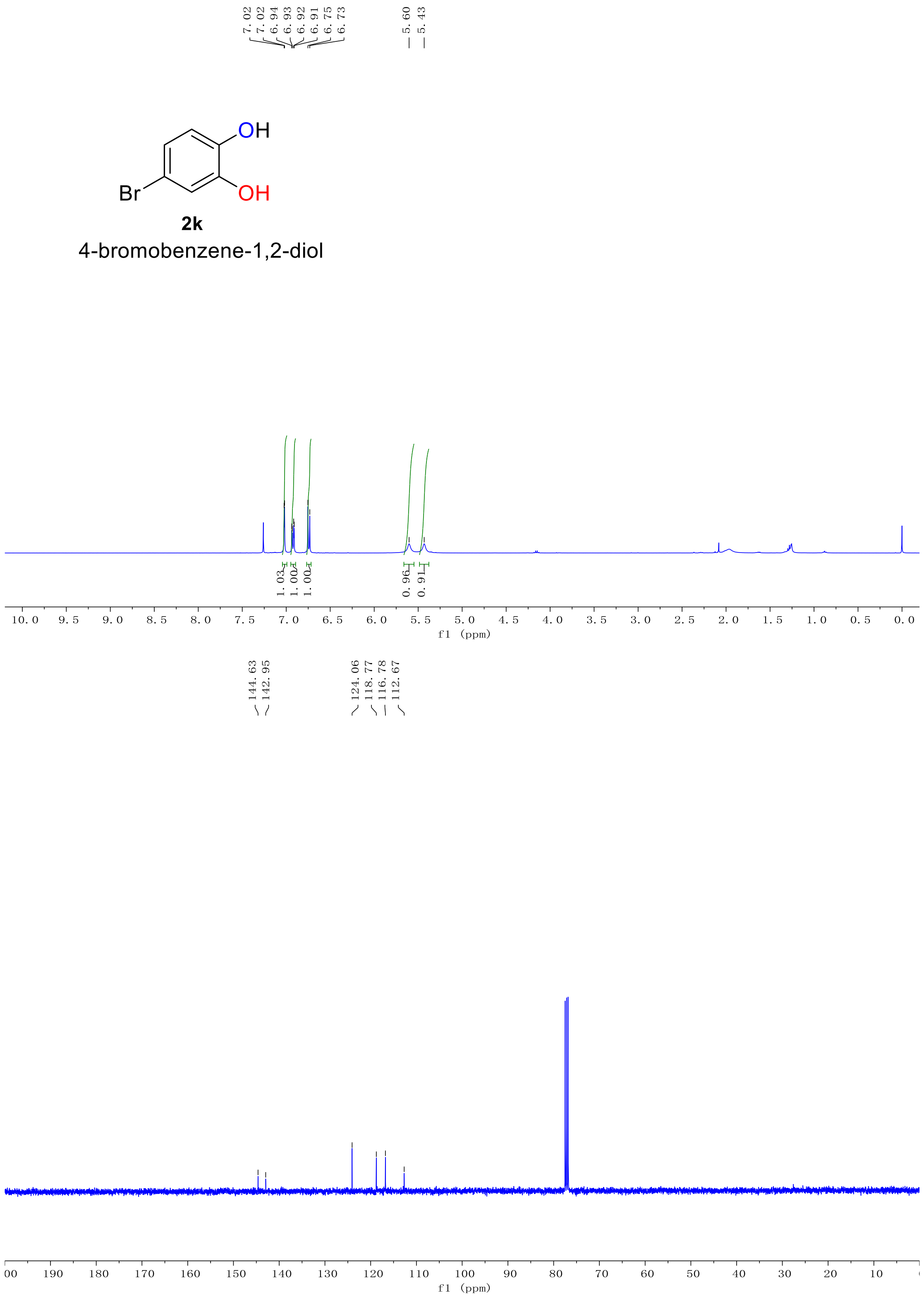

Supplementary Figure 15. ^{1}H and ^{13}C NMR spectra for compound **2k**.

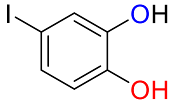

2l

4-iodobenzene-1,2-diol

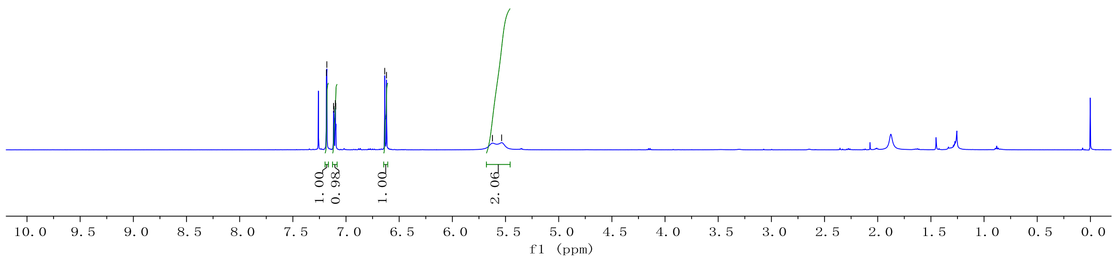

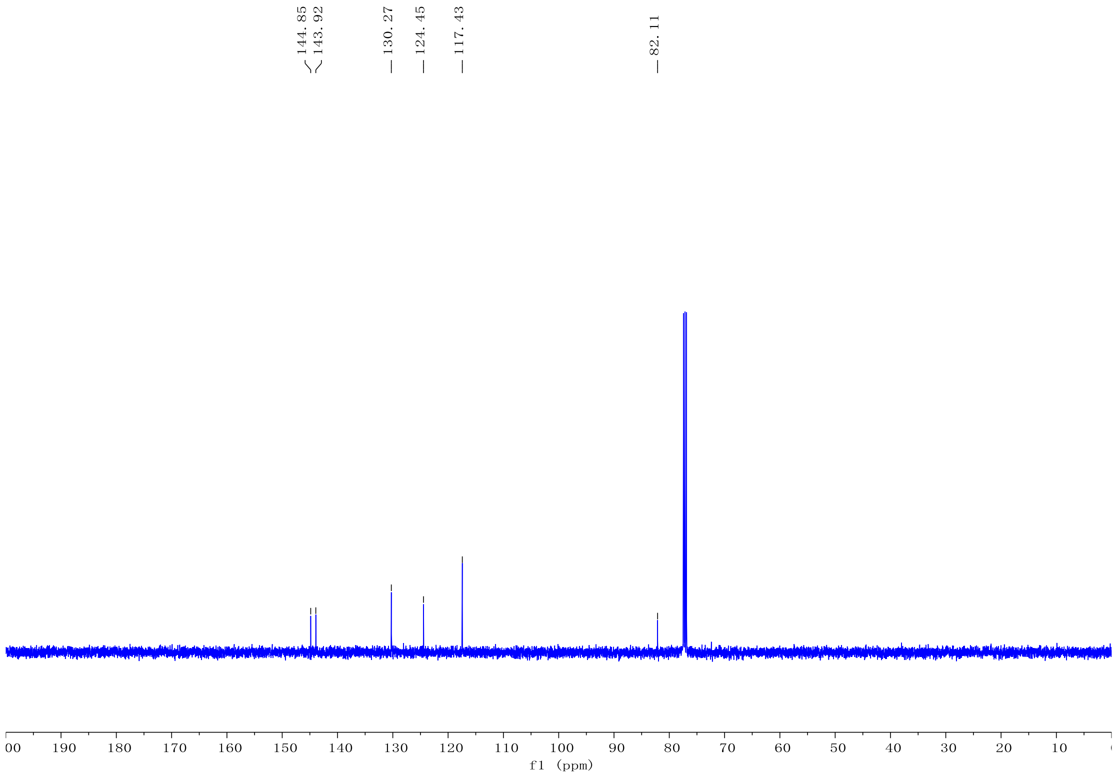

Supplementary Figure 16. ^{1}H and ^{13}C NMR spectra for compound **2l**.

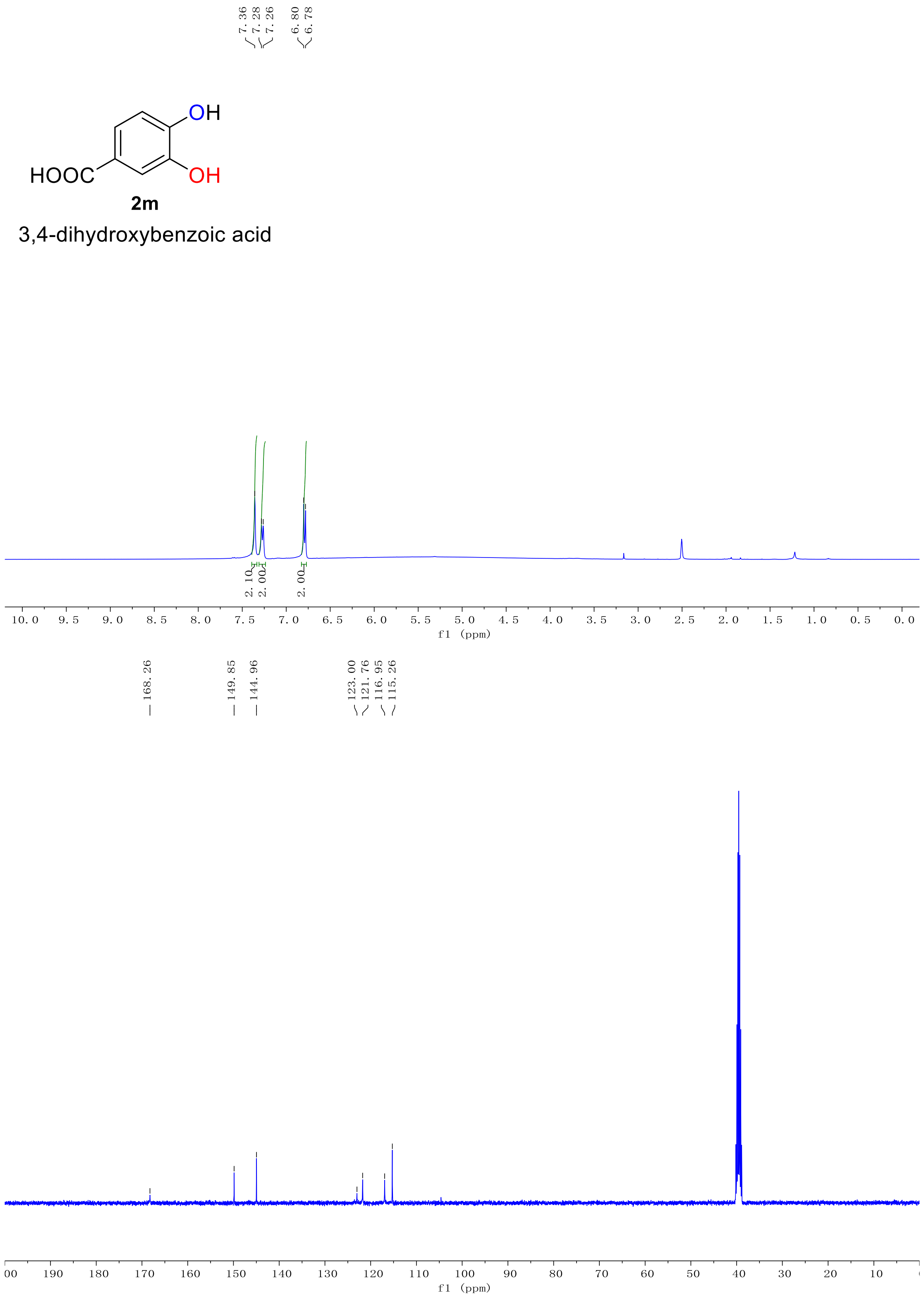

Supplementary Figure 17. ^{1}H and ^{13}C NMR spectra for compound **2m**.

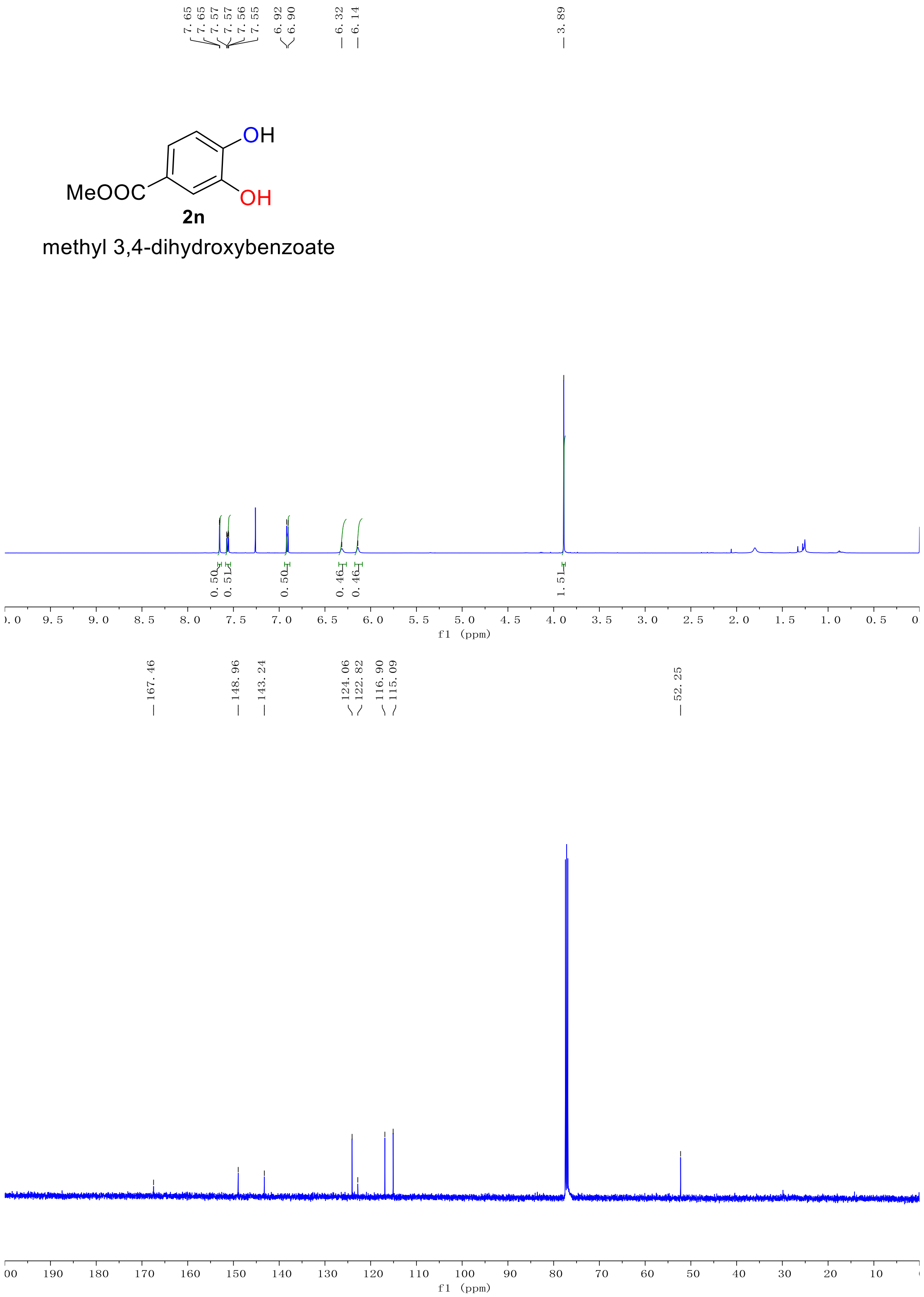

Supplementary Figure 18. ^{1}H and ^{13}C NMR spectra for compound **2n**.

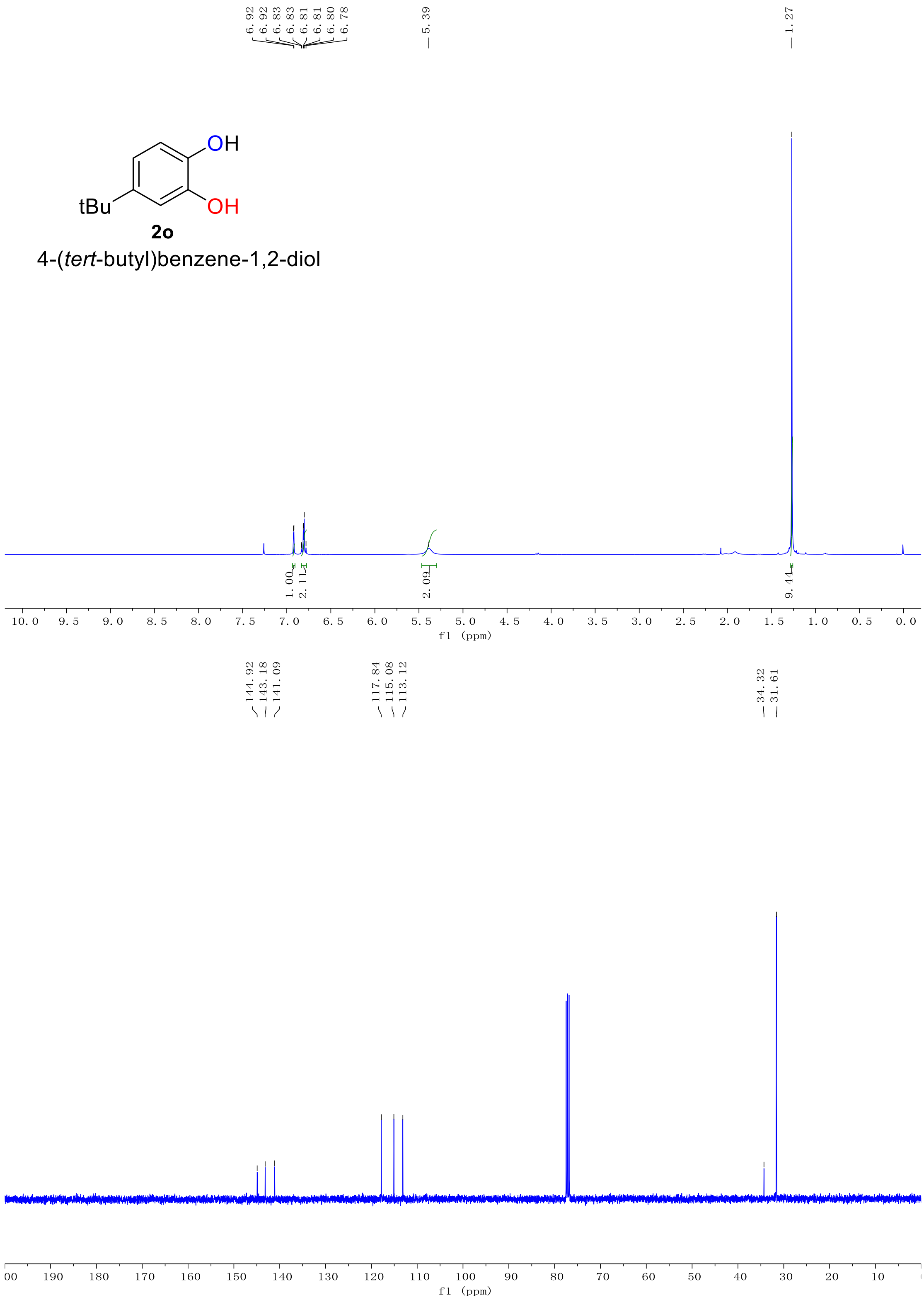

Supplementary Figure 19. ^{1}H and ^{13}C NMR spectra for compound **2o**.

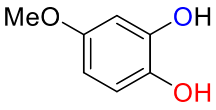

2p

4-methoxybenzene-1,2-diol

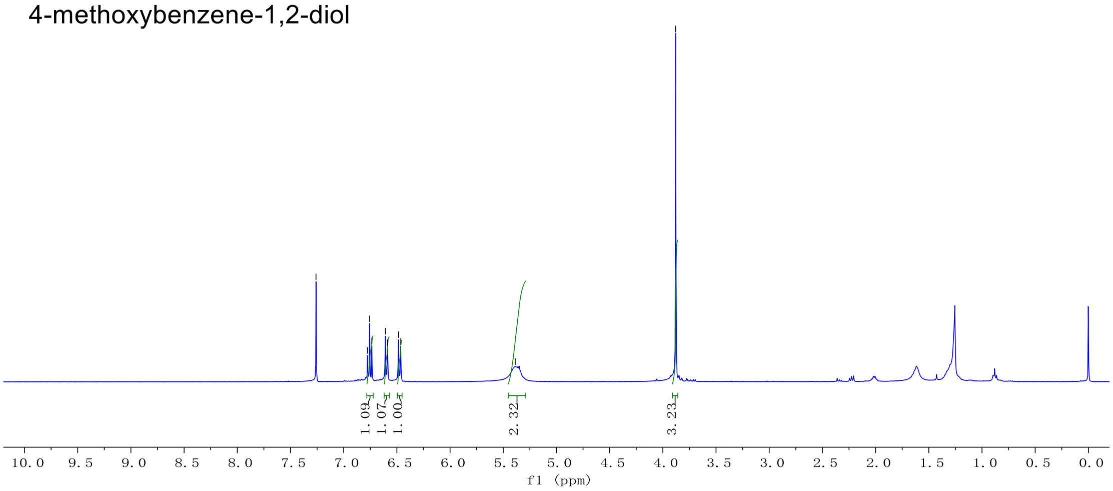

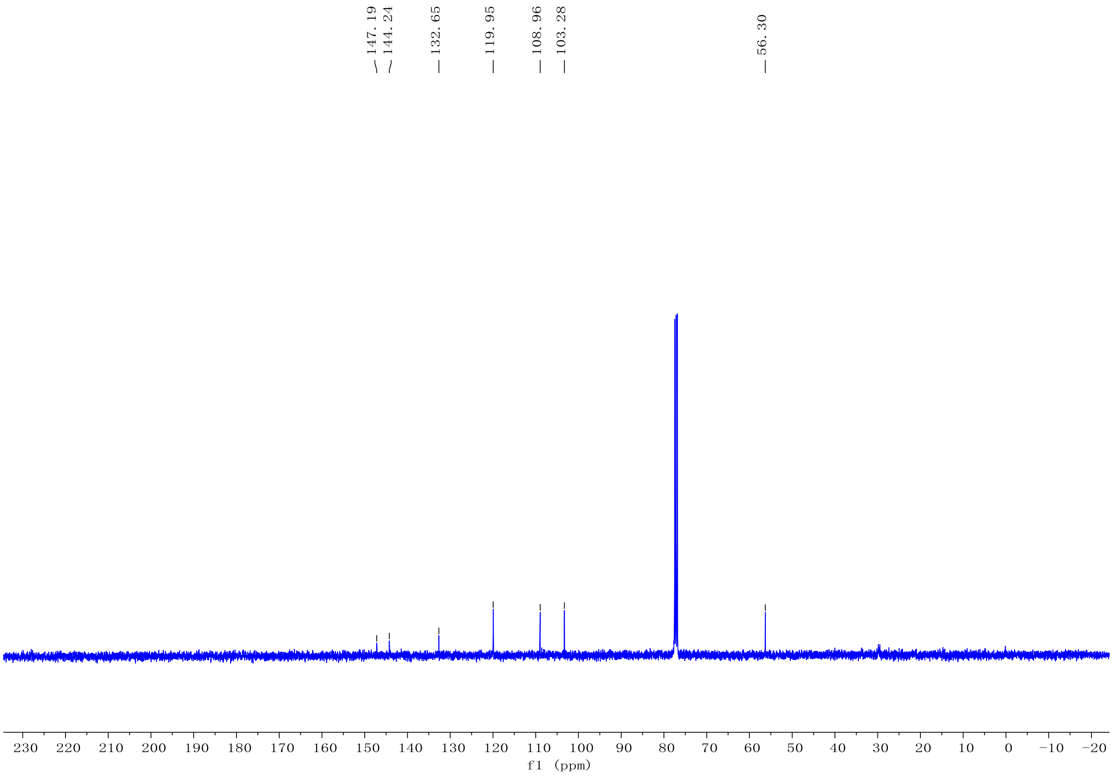

Supplementary Figure 20. ^{1}H and ^{13}C NMR spectra for compound **2p**.

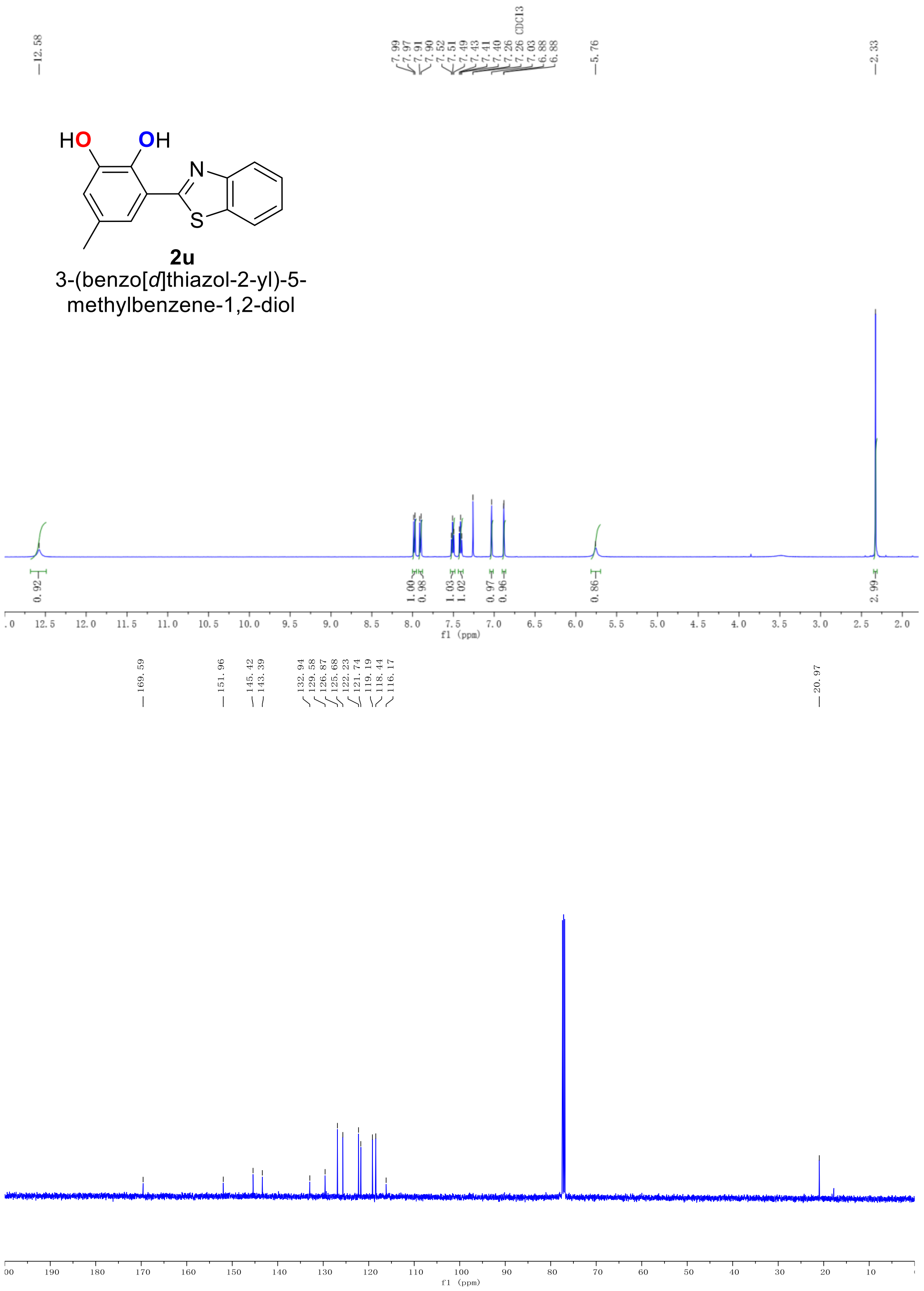

Supplementary Figure 21. ^{1}H and ^{13}C NMR spectra for compound **2u**.

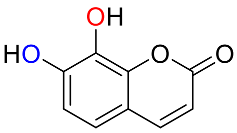

2v
7,8-dihydroxy-2*H*-chromen-2-one

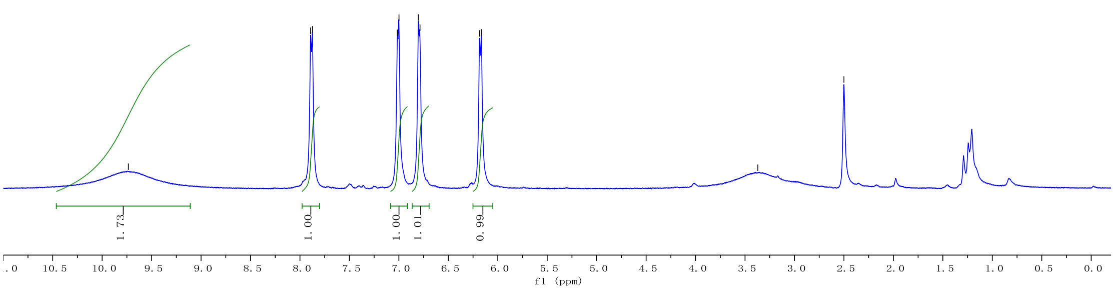

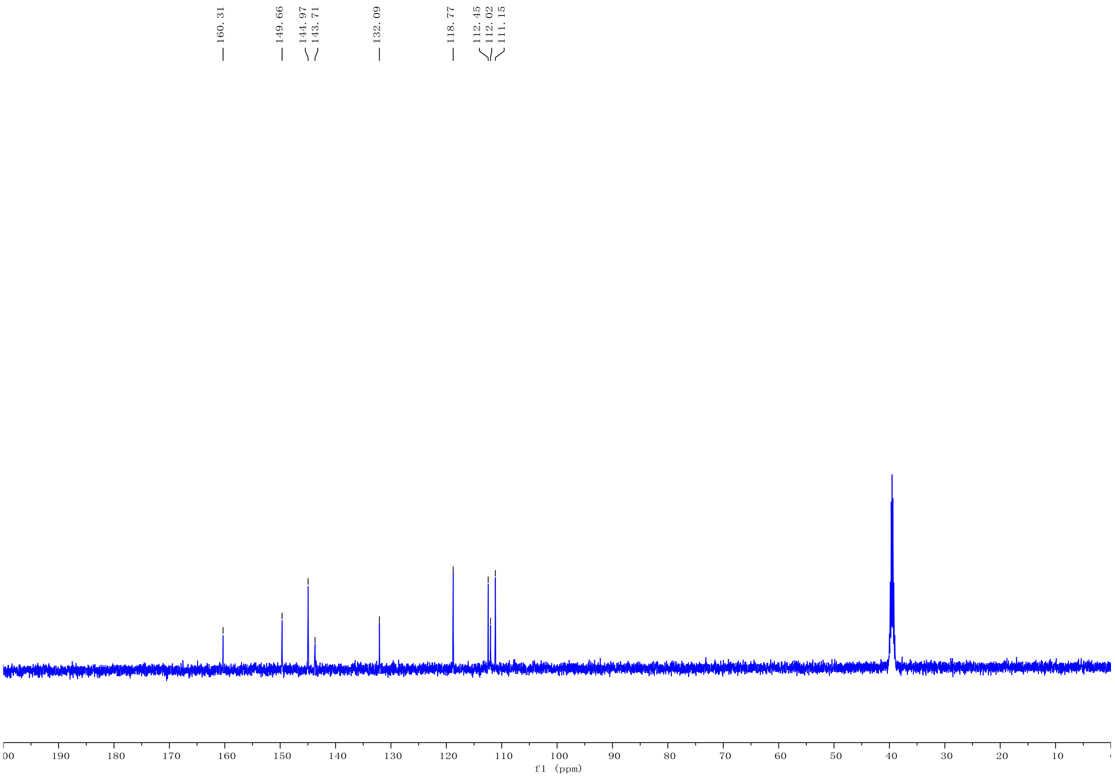

Supplementary Figure 22. ^{1}H and ^{13}C NMR spectra for compound **2v**.

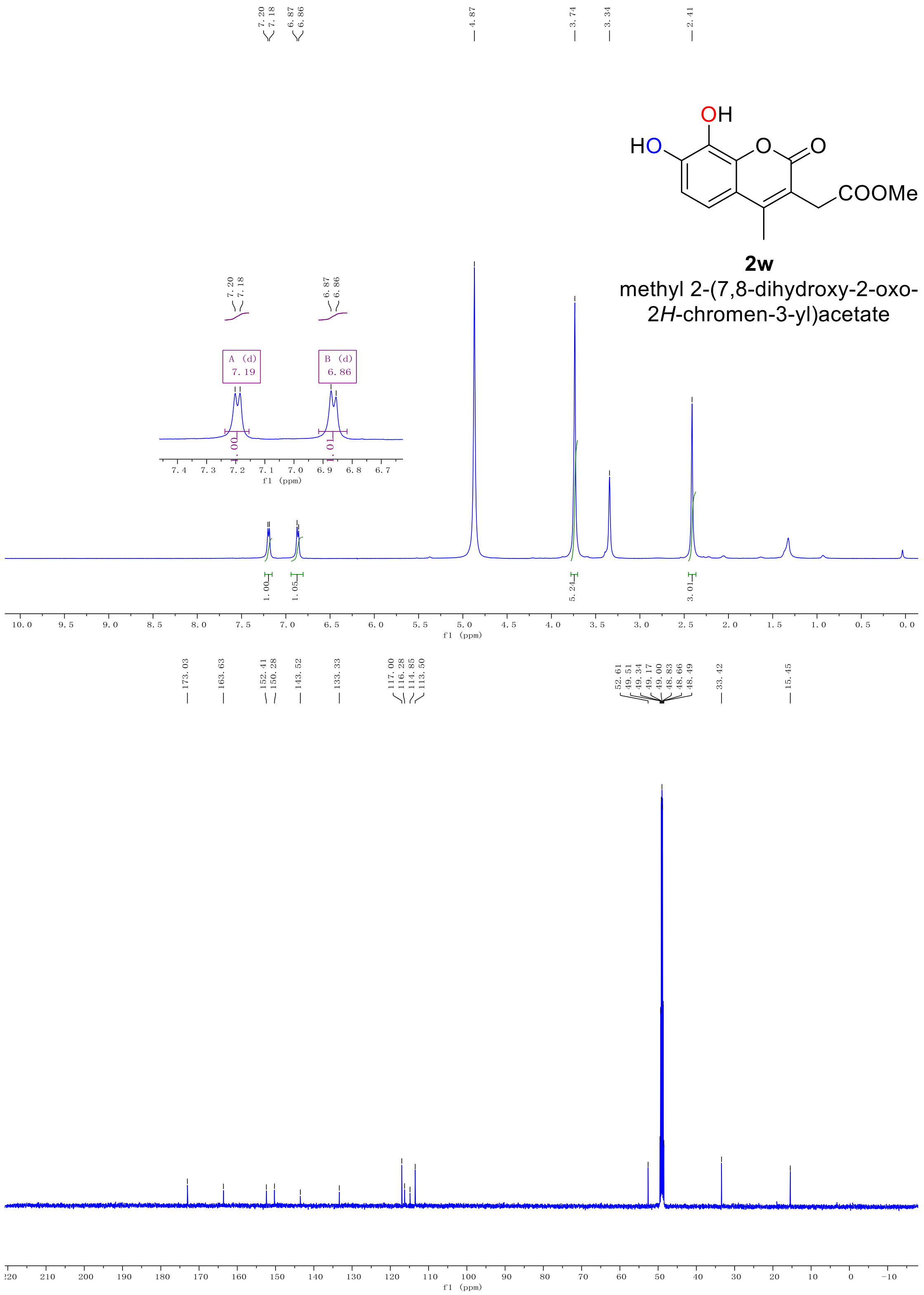

Supplementary Figure 23. ^{1}H and ^{13}C NMR spectra for compound **2w**.

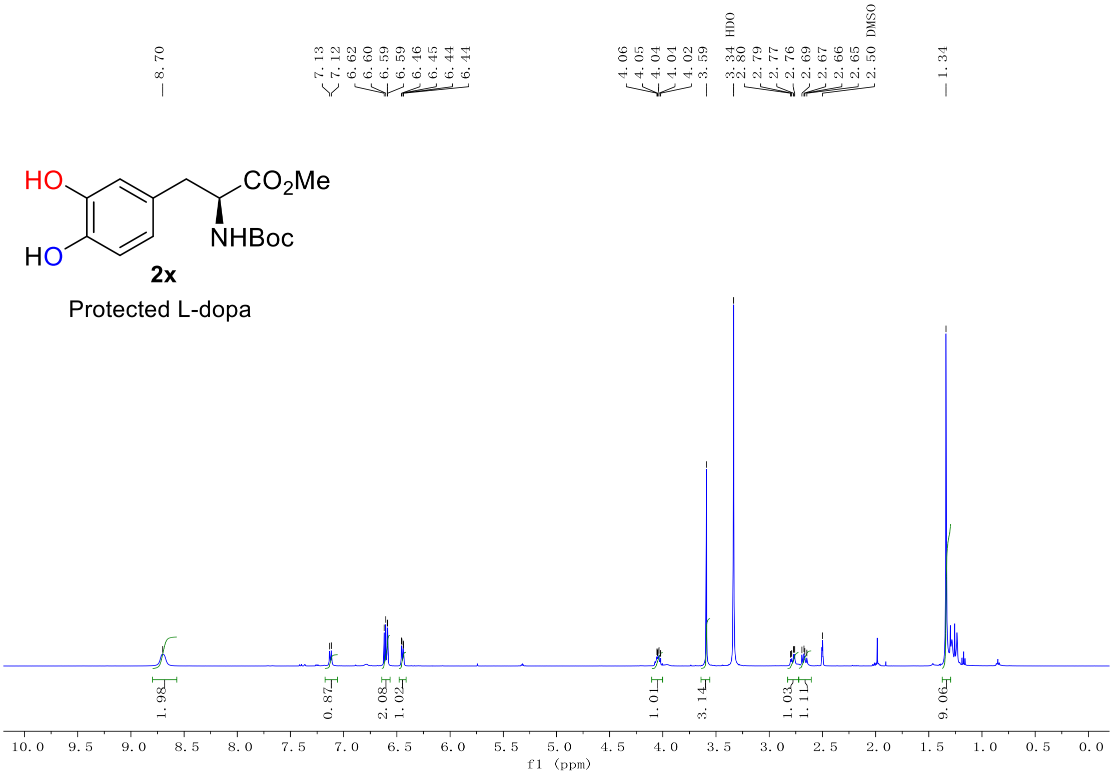

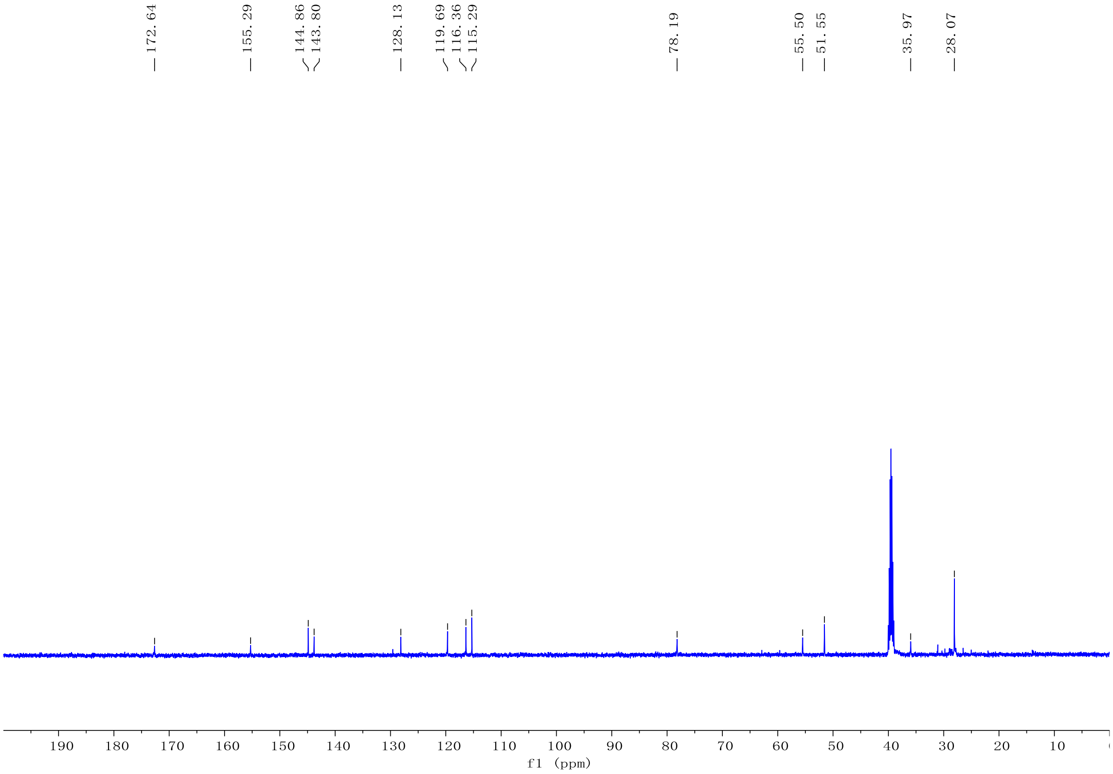

Supplementary Figure 24. ^{1}H and ^{13}C NMR spectra for compound **2x**.

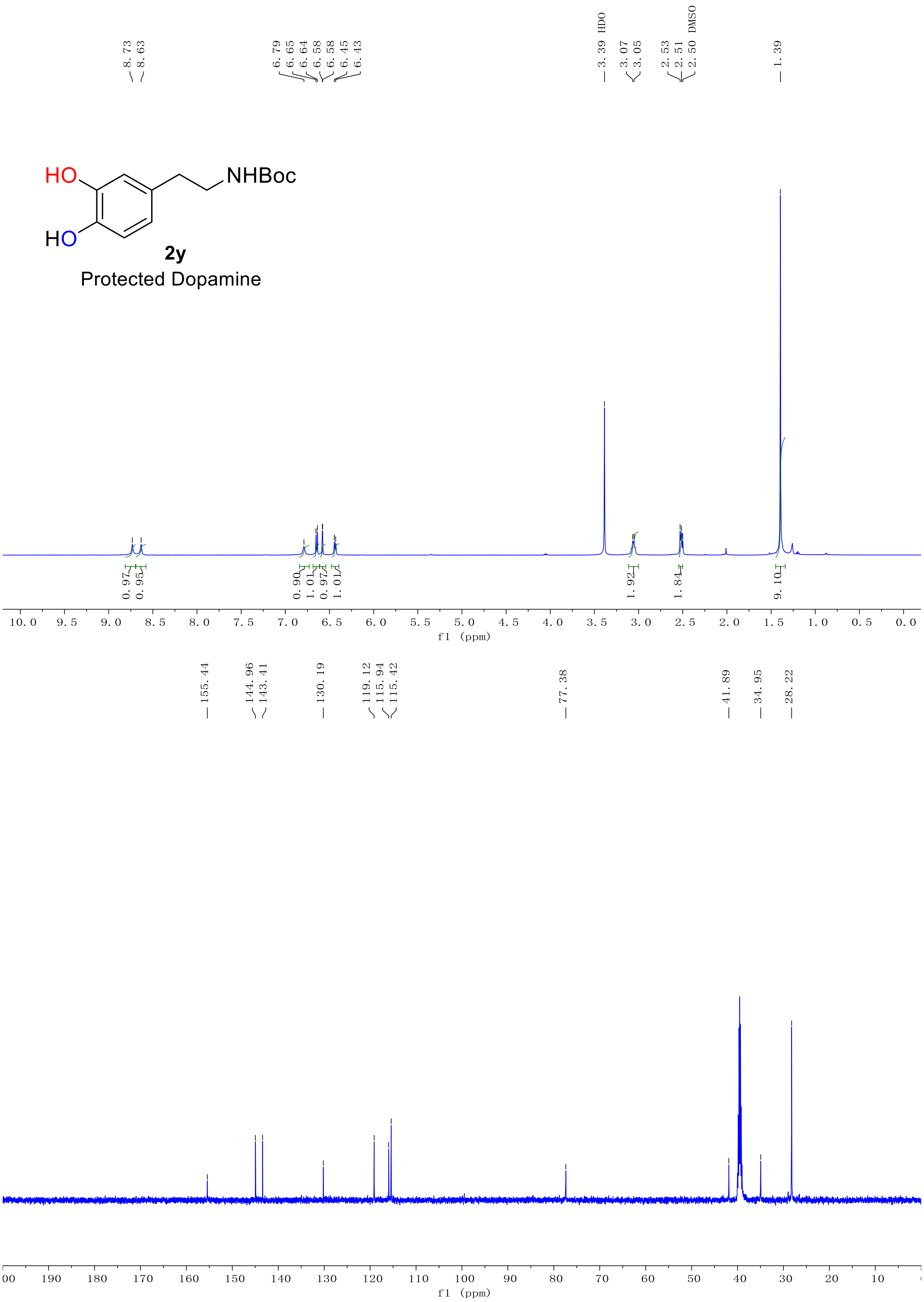

Supplementary Figure 25. ^{1}H and ^{13}C NMR spectra for compound **2y**.

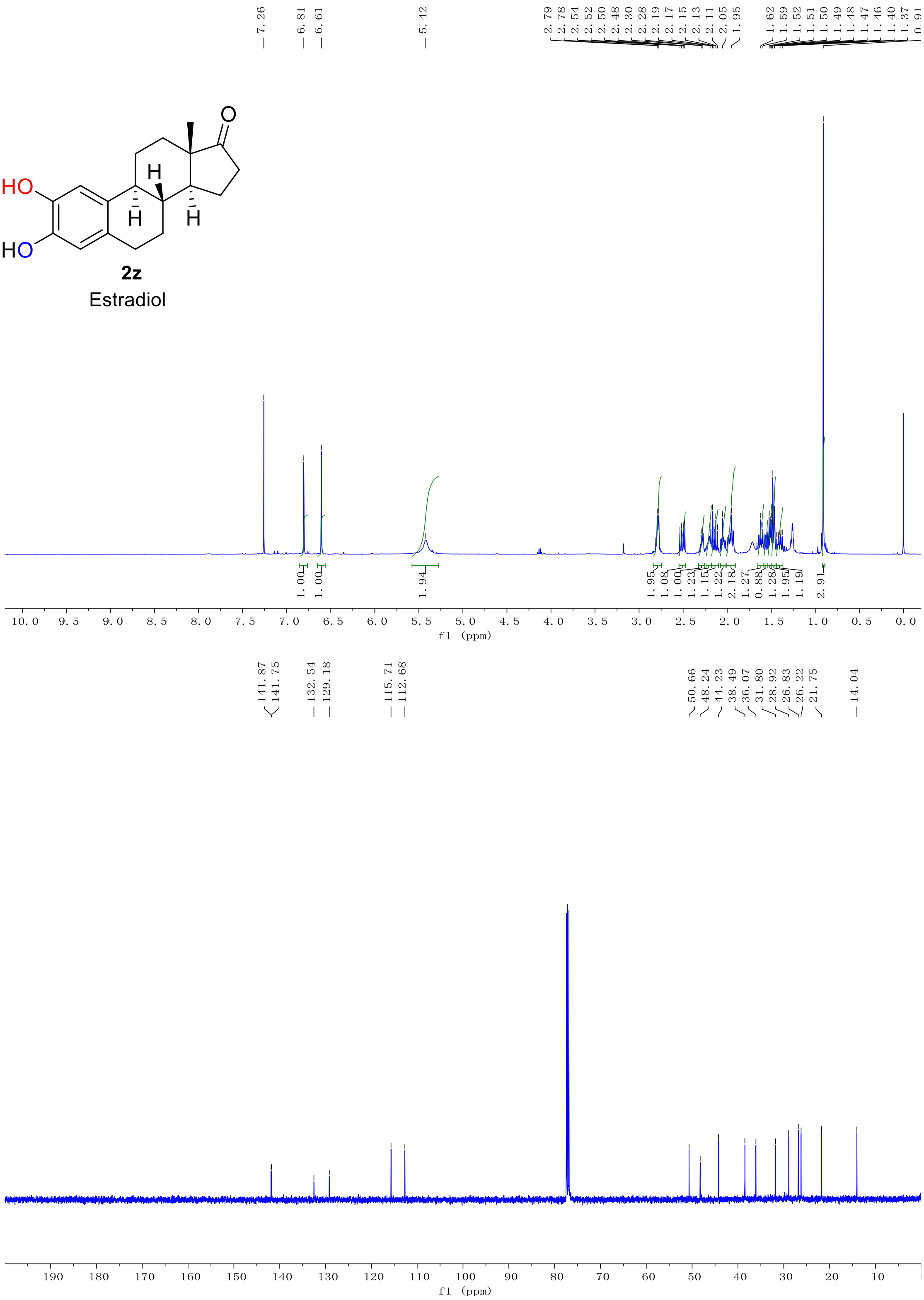

Supplementary Figure 26. ^{1}H and ^{13}C NMR spectra for compound **2z**.

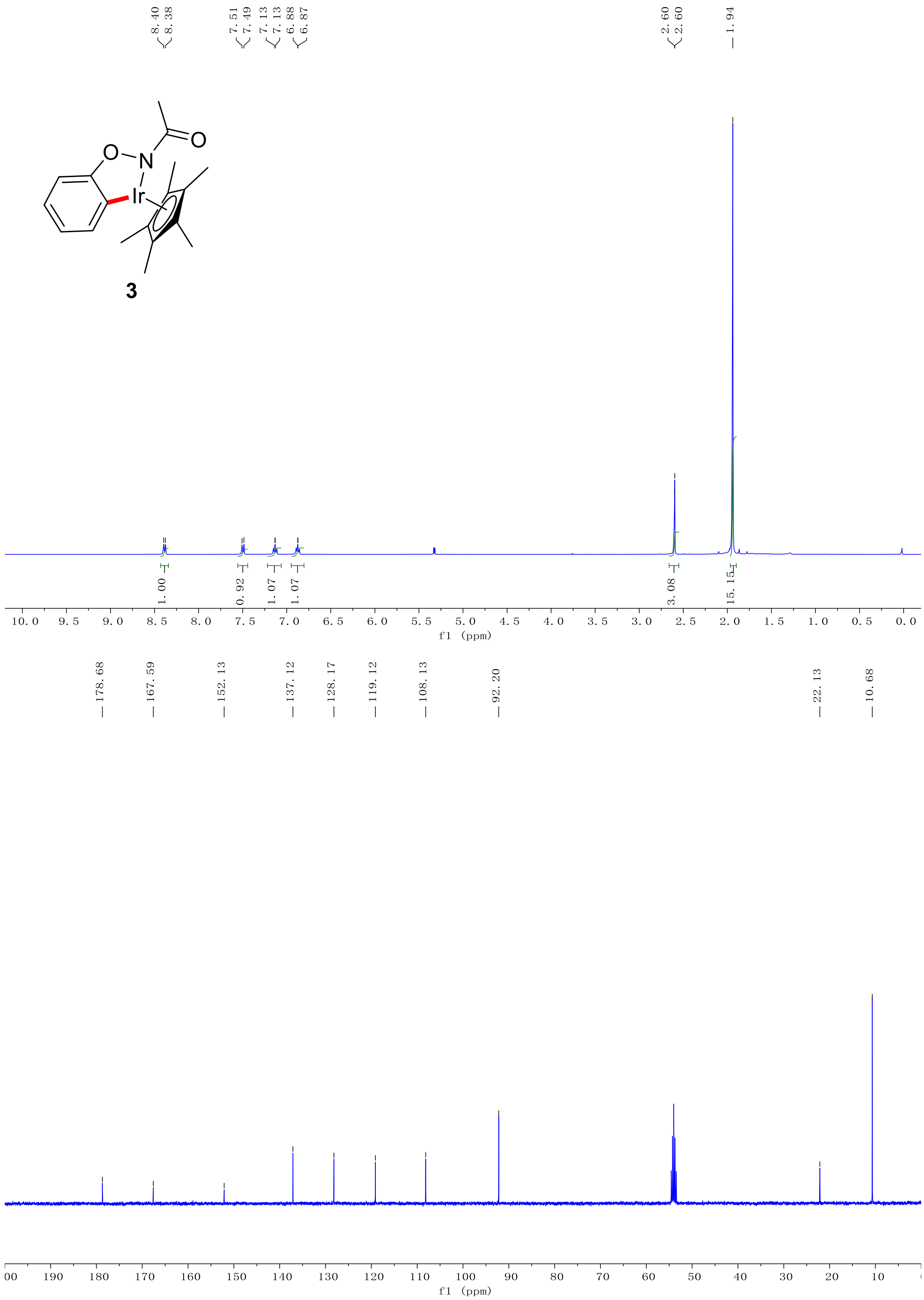

Supplementary Figure 27. ^{1}H and ^{13}C NMR spectra for compound **3**.

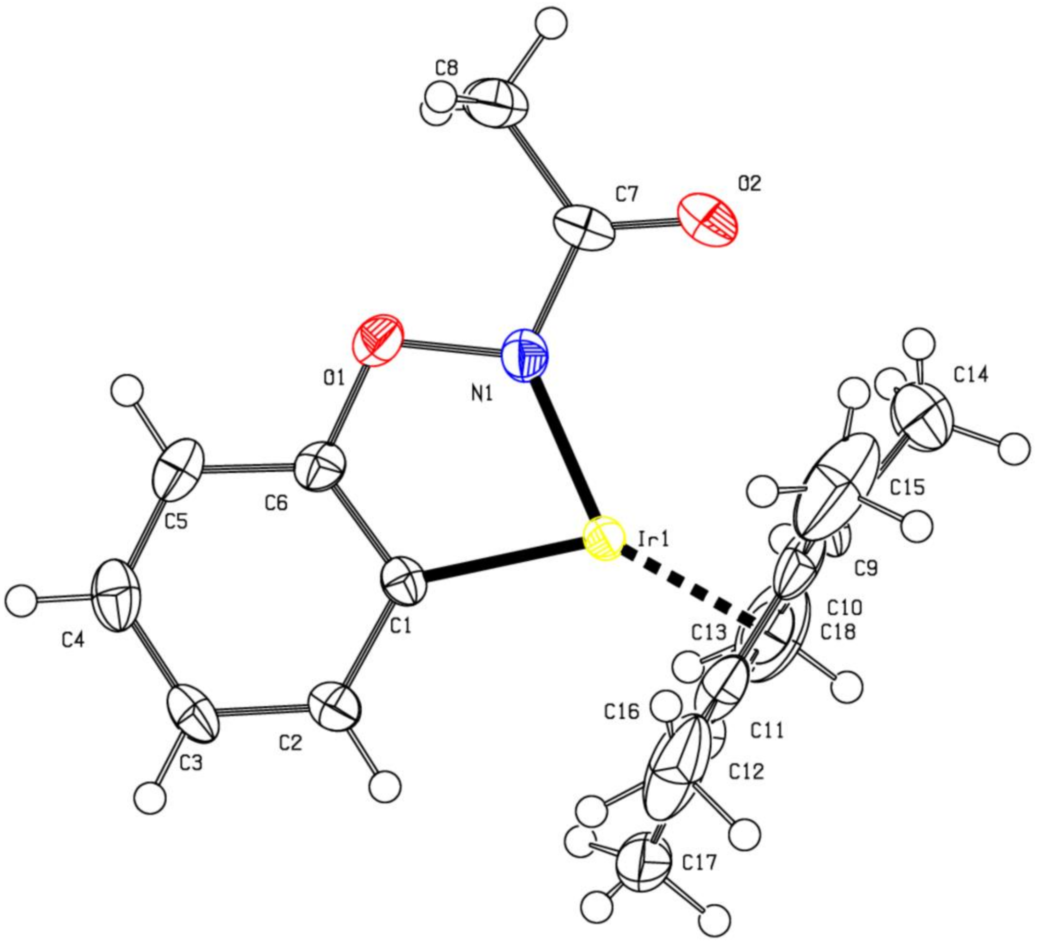

Supplementary Figure 28. Molecular structure and atom numbering scheme for **3**.

Supplementary Table

Supplementary Table 1. Crystal data and structure refinement for **3**.

Identification code	20160607zj_0m_a	
Empirical formula	C18 H22 Ir N O2	
Formula weight	476.57	
Temperature	296(2) K	
Wavelength	0.71073 Å	
Crystal system	Monoclinic	
Space group	$P2_1/c$	
Unit cell dimensions	a = 10.982(2) Å	α= 90°.
	b = 9.1215(14) Å	β= 106.645(5)°.
	c = 17.348(3) Å	γ = 90°.
Volume	1665.0(5) $Å^3$	
Z	4	
Density (calculated)	1.901 Mg/m^3	
Absorption coefficient	8.025 mm^{-1}	
F(000)	920	
Crystal size	0.28 x 0.26 x 0.04 mm^3	
Theta range for data collection	2.55 to 25.01°.	
Index ranges	-12<=h<=13, -10<=k<=10, -20<=l<=15	
Reflections collected	10981	
Independent reflections	2920 [R(int) = 0.0433]	
Completeness to theta = 25.01°	99.5 %	
Absorption correction	Semi-empirical from equivalents	
Max. and min. transmission	0.7396 and 0.2122	
Refinement method	Full-matrix least-squares on F^2	
Data / restraints / parameters	2920 / 0 / 205	
Goodness-of-fit on F^2	1.124	
Final R indices [I>2sigma(I)]	R1 = 0.0263, wR2 = 0.0820	
R indices (all data)	R1 = 0.0290, wR2 = 0.0838	
Largest diff. peak and hole	1.630 and -1.198 e.$Å^{-3}$	

Supplementary Table 2. Bond lengths [Å] and angles [°] for **3**.

C(1)-C(6)	1.394(7)
C(1)-C(2)	1.405(7)
C(1)-Ir(1)	2.010(5)
C(2)-C(3)	1.387(8)
C(2)-H(2)	0.9300
C(3)-C(4)	1.389(9)
C(3)-H(3)	0.9300
C(4)-C(5)	1.372(9)
C(4)-H(4)	0.9300
C(5)-C(6)	1.389(8)
C(5)-H(5)	0.9300
C(6)-O(1)	1.361(6)
C(7)-O(2)	1.220(7)
C(7)-N(1)	1.388(7)
C(7)-C(8)	1.505(8)
C(8)-H(8A)	0.9600
C(8)-H(8B)	0.9600
C(8)-H(8C)	0.9600
C(9)-C(10)	1.399(9)
C(9)-C(13)	1.431(9)
C(9)-C(14)	1.502(8)
C(9)-Ir(1)	2.249(5)
C(10)-C(11)	1.439(9)
C(10)-C(15)	1.488(9)
C(10)-Ir(1)	2.187(5)
C(11)-C(12)	1.426(10)
C(11)-C(16)	1.500(9)
C(11)-Ir(1)	2.139(6)
C(12)-C(13)	1.440(8)
C(12)-C(17)	1.472(9)
C(12)-Ir(1)	2.180(6)
C(13)-C(18)	1.508(9)
C(13)-Ir(1)	2.159(6)
C(14)-H(14A)	0.9600
C(14)-H(14B)	0.9600
C(14)-H(14C)	0.9600

C(15)-H(15A)	0.9600
C(15)-H(15B)	0.9600
C(15)-H(15C)	0.9600
C(16)-H(16A)	0.9600
C(16)-H(16B)	0.9600
C(16)-H(16C)	0.9600
C(17)-H(17A)	0.9600
C(17)-H(17B)	0.9600
C(17)-H(17C)	0.9600
C(18)-H(18A)	0.9600
C(18)-H(18B)	0.9600
C(18)-H(18C)	0.9600
Ir(1)-N(1)	1.952(4)
N(1)-O(1)	1.444(5)

C(6)-C(1)-C(2)	115.0(5)
C(6)-C(1)-Ir(1)	114.1(4)
C(2)-C(1)-Ir(1)	130.9(4)
C(3)-C(2)-C(1)	121.8(5)
C(3)-C(2)-H(2)	119.1
C(1)-C(2)-H(2)	119.1
C(2)-C(3)-C(4)	120.1(5)
C(2)-C(3)-H(3)	120.0
C(4)-C(3)-H(3)	120.0
C(5)-C(4)-C(3)	120.7(5)
C(5)-C(4)-H(4)	119.7
C(3)-C(4)-H(4)	119.7
C(4)-C(5)-C(6)	117.7(5)
C(4)-C(5)-H(5)	121.1
C(6)-C(5)-H(5)	121.1
O(1)-C(6)-C(5)	116.3(5)
O(1)-C(6)-C(1)	119.0(5)
C(5)-C(6)-C(1)	124.7(5)
O(2)-C(7)-N(1)	119.0(5)
O(2)-C(7)-C(8)	122.0(5)
N(1)-C(7)-C(8)	119.0(5)
C(7)-C(8)-H(8A)	109.5
C(7)-C(8)-H(8B)	109.5

H(8A)-C(8)-H(8B)	109.5
C(7)-C(8)-H(8C)	109.5
H(8A)-C(8)-H(8C)	109.5
H(8B)-C(8)-H(8C)	109.5
C(10)-C(9)-C(13)	107.3(5)
C(10)-C(9)-C(14)	126.1(7)
C(13)-C(9)-C(14)	126.5(7)
C(10)-C(9)-Ir(1)	69.2(3)
C(13)-C(9)-Ir(1)	67.7(3)
C(14)-C(9)-Ir(1)	130.1(5)
C(9)-C(10)-C(11)	108.5(5)
C(9)-C(10)-C(15)	128.0(8)
C(11)-C(10)-C(15)	123.4(8)
C(9)-C(10)-Ir(1)	74.0(3)
C(11)-C(10)-Ir(1)	68.8(3)
C(15)-C(10)-Ir(1)	123.2(4)
C(12)-C(11)-C(10)	108.8(5)
C(12)-C(11)-C(16)	124.0(7)
C(10)-C(11)-C(16)	126.8(7)
C(12)-C(11)-Ir(1)	72.3(4)
C(10)-C(11)-Ir(1)	72.4(3)
C(16)-C(11)-Ir(1)	126.2(4)
C(11)-C(12)-C(13)	105.6(5)
C(11)-C(12)-C(17)	129.6(7)
C(13)-C(12)-C(17)	124.8(7)
C(11)-C(12)-Ir(1)	69.2(3)
C(13)-C(12)-Ir(1)	69.8(3)
C(17)-C(12)-Ir(1)	127.6(4)
C(9)-C(13)-C(12)	109.5(5)
C(9)-C(13)-C(18)	124.3(6)
C(12)-C(13)-C(18)	126.2(7)
C(9)-C(13)-Ir(1)	74.5(3)
C(12)-C(13)-Ir(1)	71.4(3)
C(18)-C(13)-Ir(1)	122.7(4)
C(9)-C(14)-H(14A)	109.5
C(9)-C(14)-H(14B)	109.5
H(14A)-C(14)-H(14B)	109.5
C(9)-C(14)-H(14C)	109.5

H(14A)-C(14)-H(14C)	109.5
H(14B)-C(14)-H(14C)	109.5
C(10)-C(15)-H(15A)	109.5
C(10)-C(15)-H(15B)	109.5
H(15A)-C(15)-H(15B)	109.5
C(10)-C(15)-H(15C)	109.5
H(15A)-C(15)-H(15C)	109.5
H(15B)-C(15)-H(15C)	109.5
C(11)-C(16)-H(16A)	109.5
C(11)-C(16)-H(16B)	109.5
H(16A)-C(16)-H(16B)	109.5
C(11)-C(16)-H(16C)	109.5
H(16A)-C(16)-H(16C)	109.5
H(16B)-C(16)-H(16C)	109.5
C(12)-C(17)-H(17A)	109.5
C(12)-C(17)-H(17B)	109.5
H(17A)-C(17)-H(17B)	109.5
C(12)-C(17)-H(17C)	109.5
H(17A)-C(17)-H(17C)	109.5
H(17B)-C(17)-H(17C)	109.5
C(13)-C(18)-H(18A)	109.5
C(13)-C(18)-H(18B)	109.5
H(18A)-C(18)-H(18B)	109.5
C(13)-C(18)-H(18C)	109.5
H(18A)-C(18)-H(18C)	109.5
H(18B)-C(18)-H(18C)	109.5
N(1)-Ir(1)-C(1)	78.5(2)
N(1)-Ir(1)-C(11)	148.5(2)
C(1)-Ir(1)-C(11)	112.7(2)
N(1)-Ir(1)-C(13)	132.6(2)
C(1)-Ir(1)-C(13)	129.2(2)
C(11)-Ir(1)-C(13)	64.2(2)
N(1)-Ir(1)-C(12)	170.5(2)
C(1)-Ir(1)-C(12)	104.8(2)
C(11)-Ir(1)-C(12)	38.5(3)
C(13)-Ir(1)-C(12)	38.8(2)
N(1)-Ir(1)-C(10)	117.5(2)
C(1)-Ir(1)-C(10)	146.5(2)

C(11)-Ir(1)-C(10)	38.8(2)
C(13)-Ir(1)-C(10)	63.2(2)
C(12)-Ir(1)-C(10)	64.5(2)
N(1)-Ir(1)-C(9)	111.6(2)
C(1)-Ir(1)-C(9)	167.0(2)
C(11)-Ir(1)-C(9)	63.3(2)
C(13)-Ir(1)-C(9)	37.8(2)
C(12)-Ir(1)-C(9)	63.9(2)
C(10)-Ir(1)-C(9)	36.7(2)
C(7)-N(1)-O(1)	109.2(4)
C(7)-N(1)-Ir(1)	131.1(4)
O(1)-N(1)-Ir(1)	119.5(3)
C(6)-O(1)-N(1)	108.8(4)

Supplementary Table 3. The supplementary screen of reaction conditions.

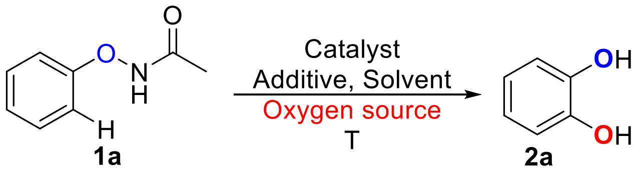

entry	catalyst[a]	additive	solvent	O source	T/℃	% yield[b]
1	$[(COD)Rh(acac)]_2$	2.5 eq. $CH_2(COOH)_2$	MeOH	—	r.t.	ND
2	$[(COD)RhCl]_2$	2.5 eq. $CH_2(COOH)_2$	MeOH	—	r.t.	ND
3	$Rh(OAc)_2$	2.5 eq. $CH_2(COOH)_2$	MeOH	—	r.t.	ND
4	$[Cp^*RhCl_2]_2$	2.5 eq. $CH_2(COOH)_2$	MeOH	—	r.t.	ND
5	$Pd(OAc)_2$	2.5 eq. $CH_2(COOH)_2$	MeOH	—	r.t.	ND
6	$Pb(acac)_2$	2.5 eq. $CH_2(COOH)_2$	MeOH	—	r.t.	ND
7	$Pb(PPh_3)_4$	2.5 eq. $CH_2(COOH)_2$	MeOH	—	r.t.	ND
8	$Pb(TFA)_2$	2.5 eq. $CH_2(COOH)_2$	MeOH	—	r.t.	ND
9	$[Cp^*IrCl_2]_2$	2.5 eq. HOOCCOOH	MeOH	—	r.t.	ND
10	$[Cp^*IrCl_2]_2$	2.5 eq. TsOH	MeOH	—	r.t.	ND
11	$[Cp^*IrCl_2]_2$	2.5 eq. $CH_2CHCOOH$	MeOH	—	r.t.	ND
12	$[Cp^*IrCl_2]_2$	2.5 eq. TMBA	MeOH	—	r.t.	63
13	$[Cp^*IrCl_2]_2$	2.5 eq. *p*-Toluic acid	MeOH	—	r.t.	46
14	$[Cp^*IrCl_2]_2$	2.5 eq. 1-adamantoic acid	MeOH	—	r.t.	54
15	$[Cp^*IrCl_2]_2$	2.5 eq. $CH_2(COOH)_2$	MeOH	—	r.t.	92
16	$[Cp^*IrCl_2]_2$	2.5 eq. $CH_2(COOH)_2$	EtOH	—	r.t.	73
17	$[Cp^*IrCl_2]_2$	2.5 eq. $CH_2(COOH)_2$	^{i}PrOH	—	r.t.	23
18	$[Cp^*IrCl_2]_2$	2.5 eq. $CH_2(COOH)_2$	AmOH	—	r.t.	ND
19	$[Cp^*IrCl_2]_2$	2.5 eq. $CH_2(COOH)_2$	Ethylene glycol	—	r.t.	15
20	$[Cp^*IrCl_2]_2$	2.5 eq. $CH_2(COOH)_2$	MeCN	—	r.t.	ND
21	$[Cp^*IrCl_2]_2$	2.5 eq. $CH_2(COOH)_2$	DMSO	—	r.t.	ND
22	$[Cp^*IrCl_2]_2$	2.5 eq. $CH_2(COOH)_2$	THF	—	r.t.	ND

a Reaction conditions: 0.2 mmol **1a**, 5 mol% catalyst, 5.0 mmol acid in 0.1 M solvent at room temperature under N_2 for 12 h. ***b*** GC yield.

Supplementary Methods

General

All reactions were carried out in air unless otherwise noted. Reaction temperatures are reported as those of the oil bath. The dry solvents used were purified by distillation and were transferred under nitrogen.

Commercially available chemicals were obtained from Sigma-Aldrich, Alfa Aesar, TCI and Aladdin and used as received unless otherwise stated. Dichloro (η^5-pentamethylcyclopentadienyl) Iridium (III) dimer (99%) was purchased from Sinocompound Catalysts Co., Ltd.

Reactions were monitored with analytical thin-layer chromatography (TLC) on silica. ^{1}H NMR and ^{13}C NMR data were recorded on Bruker nuclear resonance (400 MHz) spectrometers unless otherwise specified, respectively. Chemical shifts (δ) are given in ppm relative to TMS. The residual solvent signals were used as references and the chemical shifts converted to the TMS scale ($CDCl_3$: δ_H=7.26 ppm, δ_c=77.16 ppm; CD_2Cl_2: δ_H=5.32 ppm, δ_c=53.84 ppm; DMSO: δ_H=2.50 ppm, δ_c=39.52 ppm). HRMS (ESI) analysis was performed by The Analytical Instrumentation Center at University, Shenzhen Graduate School and (HRMS) data were reported with ion mass/charge (m/z) ratios as values in atomic mass units.

Preparation of substrates 1

Synthesis of Substrates 1:

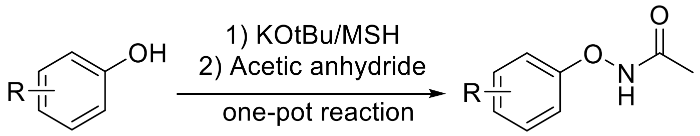

General procedure A:

Following literature reports[1,2], Phenols (1 eq.) was dissolved in 4 mL of methanol, and then potassium *tert*-butoxide (1 eq.) was added. The mixture was allowed to stir for 0.5 h under N_2 atmosphere. The methanol was removed, and the residue was taken up in 2 mL of dichloromethane. Then the freshly prepared *O*-mesitylsulfonylhydroxyl-amine (378 mg, 1.76 mmol) in dichloromethane (0.2 M) was added under ice cooling. The mixture was allowed to stir for 1 h, dichloromethane was then removed under reduce pressure to afford the corresponding *N*-aryloxyamine.

In a 20 mL round-bottom flask, *N*-aryloxyamine (1.0 eq.) was dissolved in ether (0.2 M).The flask was cooled in an ice bath, to which acetic anhydride (2.0 eq.) was slowly added. The ice bath was allowed to warm to room temperature and the mixture was stirred for 3 h at room temperature. The reaction mixture was concentrated under reduced pressure and purified by flash silica gel column chromatography to give the corresponding *N*-phenoxyacetamide.

In our work, most of substrate 1 were synthesized by general procedure A.

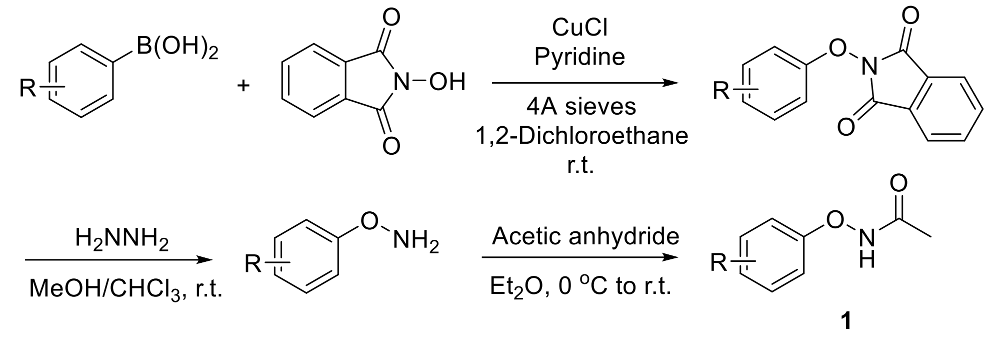

General procedure B:

Following literature reports[3,4], in a 50mL round-bottom flask, *N*-hydroxy - phthalimide (1.0 eq.), cooper (I) chloride (1.0 eq.), freshly activated 4 Å molecular sieves (250 mg/mmol), and phenylboronic acid (2.0 eq.) were combined in 1,2-dichloroethane (0.2 M). The pyridine (1.1 eq.) was then added to the suspension. The reaction mixture was open to the atmosphere and stirred at room temperature over 24-48 h. Upon completion, silica gel was added to the flask and the solvent was removed under vacuum. The desired *N*-aryloxyphthalimides were obtained by flash column chromatography on silica gel.

Hydrazine monohydrate (3.0 eq.) was added to the solution of *N*-aryloxyphth - alimide (1.0 eq.) in 10% MeOH in $CHCl_3$ (0.1 M). The reaction was allowed to stir at room temperature over 12 h. Upon completion, the reaction mixture was filtered off and washed with CH_2Cl_2. The filtrate was concentrated under reduced pressure, and purified by flash silica gel column chromatography to give the corresponding *N*-aryloxyamine.

In a 20 mL round-bottom flask, *N*-aryloxyamine (1.0 eq.) was dissolved in ether (0.2 M).The flask was cooled in an ice bath, to which acetic anhydride (2.0 eq.) was slowly added. The ice bath was allowed to warm to room temperature and the mixture was stirred for 3 h at room temperature. The reaction mixture was concentrated under reduced pressure and purified by flash silica gel column chromatography to give the corresponding *N*-phenoxyacetamide.

Characterization of substrates:

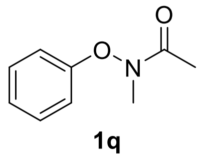

1q
N-methyl-*N*-phenoxyacetamide

The title compound was obtained in 73% yield as white solid; ^1H NMR (400 MHz, $CDCl_3$) δ 7.38 – 7.31 (m, 2H), 7.08 (t, J = 7.4 Hz, 1H), 6.98 (dd, J = 8.7, 0.8 Hz, 2H), 3.28 (s, 3H), 2.08 (s, 3H). ^{13}C NMR (101 MHz, $CDCl_3$) δ 173.35, 157.55, 130.03, 123.39, 112.90, 34.05, 20.37. HRMS (ESI): Calcd. for C9H12NO2 (M+H) 166.0868; Found: 166.0863

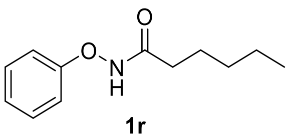

1r
N-phenoxyhexanamide

The title compound was obtained in 76% yield as white solid; ^1H NMR (400 MHz, MeOD) δ 7.31 (t, J = 7.9 Hz, 2H), 7.04 (t, J = 7.0 Hz, 3H), 4.59 (s, 1H), 2.26 (t, J = 7.4 Hz, 2H), 1.75–1.63 (m, 2H), 1.44–1.33 (m, 4H), 0.95 (t, J = 6.3 Hz, 3H). ^{13}C NMR (101 MHz, MeOD) δ 173.76, 160.97, 130.48, 123.87, 114.14, 33.61, 32.45, 26.22, 23.34, 14.25. HRMS (ESI): Calcd. for C12H18NO2 (M+H) 208.1338; Found: 208.1332.

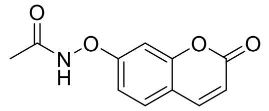

1v
N-((2-oxo-2*H*-chromen-7-yl)oxy)acetamide

The title compound was obtained in 53% yield as white solid; ^{1}H NMR (500 MHz, MeOD) :δ 7.90 (d, *J* = 9.5 Hz, 1H), 7.60 (d, *J* = 8.5 Hz, 1H), 7.11 – 6.97 (m, 2H), 6.31 (d, *J* = 9.5 Hz, 1H), 2.07 (s, 3H). ^{13}C NMR (126 MHz, MeOD) :δ 164.19, 162.86, 156.78, 145.36, 130.72, 115.77, 114.63, 111.80, 101.77, 19.30. HRMS (ESI): Calcd. for C11H10NO4 (M+H) 220.0610; Found: 220.0612.

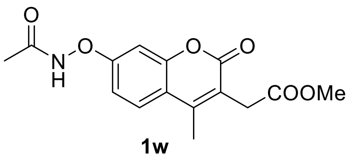

1w
methyl 2-(7-(acetamidooxy)-4-methyl-2-oxo-2*H*-chromen-3-yl)acetate

The title compound was obtained in 56% yield as white solid; ^{1}H NMR (500 MHz, DMSO-d6) δ 11.93 (s, 1H), 7.79 (d, J = 8.8 Hz, 1H), 7.07 (d, J = 7.3 Hz, 2H), 3.68 (s, 2H), 3.62 (s, 3H), 2.39 (s, 3H), 1.96 (s, 3H). ^{13}C NMR (126 MHz, DMSO) δ 170.37, 167.56, 162.02, 160.53, 152.98, 149.16, 126.94, 116.77, 114.73, 110.11, 100.16, 51.73, 32.35, 19.33, 15.03. HRMS (ESI): Calcd. for C15H16NO6 (M+H) 306.0978; Found: 306.0966.

The synthesis of catechols via Ir-catalyzed *ortho*-hydroxylation from *N*-phenoxyacetamide

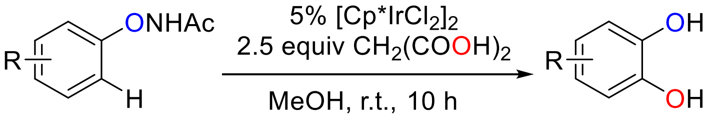

1 **2**

General procedure:

N-phenoxyacetamide (**1**) (0.2 mmol), $[Cp^*IrCl_2]_2$ (5 mol%), $CH_2(COOH)_2$ (5.0 mmol) without external oxidant were weighed into a 10 mL pressure tube, to which was added anhydrous MeOH (1 mL) in a glove box. The reaction vessel was stirred at room temperature for 10 h in air. Then the mixture was concentrated under vacuum and the residue was purified by column chromatography on silica gel with a gradient eluent of petroleum ether and ethyl acetate to afford the corresponding product.

Characterization of products:

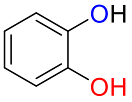

2a
pyrocatechol

The title compound was obtained in 90% yield as white solid; ^{1}H NMR (400 MHz, $CDCl_3$) δ 6.88-6.91 (m, 2H), 6.86-6.82 (m, 2H), 5.42 (s, 2H). ^{13}C NMR (101 MHz, $CDCl_3$) δ 143.51, 121.30, 115.54. HRMS (ESI): Calcd. for C13H9NOS (M-H)109.0290; Found: 109.0293.

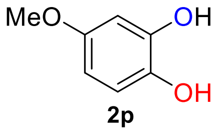

2p
4-methoxybenzene-1,2-diol

The title compound was obtained in 90% yield as white solid; ^{1}H NMR (500 MHz, Chloroform-d) δ 6.71 (s, 3H), 5.19 (s, 2H), 2.26 (s, 3H). ^{13}C NMR (126 MHz, $CDCl_3$) δ 142.99, 142.06, 124.41, 122.85, 120.07, 112.89, 15.32. HRMS (ESI): Calcd. for C7H8O2 (M-H): 123.0446; Found: 123.0450.

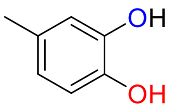

2c
4-methylbenzene-1,2-diol

The title compound was obtained in 90% yield as white solid; ^{1}H NMR (400 MHz, Chloroform-*d*) δ 6.76 (d, *J* = 8.0 Hz, 1H), 6.69 (d, *J* = 1.5 Hz, 1H), 6.63 – 6.57 (m, 1H), 5.70 (s, 2H), 2.23 (s, 3H). ^{13}C NMR (101 MHz, $CDCl_3$) δ 147.19, 144.24, 132.65, 119.95, 108.96, 103.28, 56.30. HRMS (ESI): Calcd. for C7H8O2 (M-H): 123.0446; Found: 123.0454.

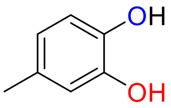

2d
4-methylbenzene-1,2-diol

The title compound was obtained in 95% yield as white solid; ^{1}H NMR (400 MHz, Chloroform-d) δ 6.76 (d, J = 8.0 Hz, 1H), 6.70 (s, 1H), 6.61 (d, J = 8.0 Hz, 1H), 5.41 (d, J = 47.7 Hz, 2H), 2.24 (s, 3H).^{13}C NMR (101 MHz, CDCl3) δ 143.46, 141.19, 131.16, 121.56, 116.37, 115.44, 20.82. HRMS (ESI): Calcd. for C7H8O2 (M-H):123.0446; Found: 123.0452.

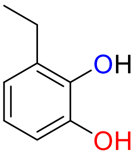

2e
3-ethylbenzene-1,2-diol

The title compound was obtained in 88% yield as white solid; ^{1}H NMR (400 MHz, Chloroform-d) δ 6.76 (d, J = 8.0 Hz, 1H), 6.69 (d, J = 1.5 Hz, 1H), 6.63 – 6.57 (m, 1H), 5.70 (s, 2H), 2.23 (s, 3H). ^{13}C NMR (101 MHz, $CDCl_3$) δ 143.11, 141.89, 130.89, 121.41, 120.43, 113.09, 22.90, 14.11. HRMS (ESI): Calcd. for C8H10O2 (M-H): 137.0603; Found: 137.0609.

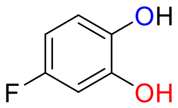

2f
4-fluorobenzene-1,2-diol

The title compound was obtained in 68% yield as white solid; ^{1}H NMR (400 MHz, Chloroform-d) δ 6.80 (dd, J = 8.8, 5.3 Hz, 1H), 6.66 (dd, J = 9.2, 2.9 Hz, 1H), 6.52 (td, J = 8.5, 2.9 Hz, 1H), 5.70 (s, 1H), 5.22 (s, 1H). ^{13}C NMR (75 MHz, Chloroform-d) δ 158.80 , 155.64 , 144.59 , 144.43 , 139.41 , 139.38 , 115.57 , 115.44 , 106.87 , 106.56 , 103.54 , 103.18. ^{19}F NMR (376 MHz, $CDCl_3$) δ -121.58. 19F NMR (376 MHz, $CDCl_3$) δ -121.58. HRMS (ESI): Calcd. for C6H5FO2 (M-H): 127.0195; Found: 127.0201.

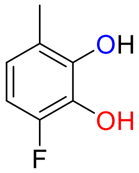

2g
3-fluoro-6-methylbenzene-1,2-diol

The title compound was obtained in 75% yield as white solid; ^{1}H NMR (400 MHz, Chloroform-*d*) δ 6.67 – 6.52 (m, 2H), 5.48 (s, 1H), 5.34 (s, 1H), 2.21 (s, 3H). ^{13}C NMR (101 MHz, $CDCl_3$) δ 150.96, 148.65, 143.71, 130.97, 130.81, 121.31, 121.23, 120.40, 106.54, 106.36, 15.30. ^{19}F NMR (376 MHz, $CDCl_3$) δ -142.30. HRMS (ESI): Calcd. for C7H7FO2 (M-H): 141.0352; Found: 141.0357.

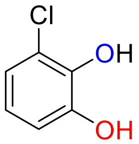

2h
3-chlorobenzene-1,2-diol

The title compound was obtained in 73% yield as white solid; ^{1}H NMR (400 MHz, Chloroform-*d*) δ 6.89 (s, 1H), 6.78 (d, *J* = 1.0 Hz, 2H), 5.44 (s, 1H), 5.24 (s, 1H). ^{13}C NMR (101 MHz, $CDCl_3$) δ 144.37, 142.48, 125.79, 121.07, 116.27, 116.00. HRMS (ESI): Calcd. for C6H5ClO2 (M-H): 143.9900; Found: 142.9905.

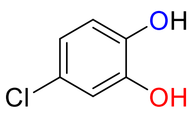

2i
4-chlorobenzene-1,2-diol

The title compound was obtained in 78% yield as white solid; ^{1}H NMR (400 MHz, Chloroform-*d*) δ 6.88 (s, 1H), 6.78 (s, 2H), 5.47 (s, 2H). ^{13}C NMR (101 MHz, $CDCl_3$) δ 144.38, 142.38, 125.75, 121.06, 116.27, 115.99. HRMS (ESI): Calcd. for C6H5ClO2 (M-H): 143.9900; Found: 142.9904.

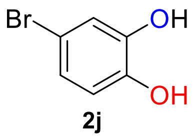

2j
4-bromobenzene-1,2-diol

The title compound was obtained in 75% yield as white solid; ^{1}H NMR (500 MHz, Chloroform-*d*) δ 7.02 (d, *J* = 2.2 Hz, 1H), 6.92 (dd, *J* = 8.5, 2.2 Hz, 1H), 6.74 (d, *J* = 8.5 Hz, 1H), 5.61 (s, 1H), 5.43 (s, 1H). ^{13}C NMR (126 MHz, $CDCl_3$) δ 144.74, 143.05, 124.06, 118.83, 116.81, 112.69. HRMS (ESI): Calcd. for C6H5BrO2 (M-H): 186.9395; Found: 186.9401, 188.9377.

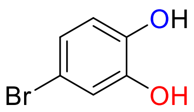

2k
4-bromobenzene-1,2-diol

The title compound was obtained in 70% yield as white solid; ^{1}H NMR (400 MHz, Chloroform-*d*) δ 7.02 (d, *J* = 2.2 Hz, 1H), 6.92 (dd, *J* = 8.5, 2.2 Hz, 1H), 6.74 (d, *J* = 8.5 Hz, 1H), 5.60 (s, 1H), 5.43 (s, 1H). ^{13}C NMR (101 MHz, $CDCl_3$) δ 144.63, 142.95, 124.06, 118.77, 116.78, 112.67. HRMS (ESI): Calcd. for C6H5BrO2 (M-H): 186.9395; Found: 186.9400, 188.9378.

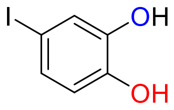

2l
4-iodobenzene-1,2-diol

The title compound was obtained in 80% yield as white solid; ^{1}H NMR (500 MHz, Chloroform-*d*) δ 7.18 (d, *J* = 2.0 Hz, 1H), 7.11 (dd, *J* = 8.3, 2.0 Hz, 1H), 6.63 (d, *J* = 8.3 Hz, 1H), 5.58 (d, *J* = 43.4 Hz, 2H). ^{13}C NMR (126 MHz, $CDCl_3$) δ 144.85, 143.92, 130.27, 124.45, 117.43, 82.11. HRMS (ESI): Calcd. for C6H5IO2 (M-H): 234.9256; Found: 234.9261

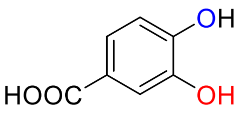

2m
3,4-dihydroxybenzoic acid

The title compound was obtained in 90% yield as white solid; ^{1}H NMR (400 MHz, DMSO-d_6) δ 7.36 (s, 2H), 7.27 (d, *J* = 8.0 Hz, 2H), 6.79 (d, *J* = 8.1 Hz, 2H). ^{13}C NMR (101 MHz, DMSO) δ 168.26, 149.85, 144.96, 123.00, 121.76, 116.95, 115.26. HRMS (ESI): Calcd. for C7H6O4 (M-H): 153.0188; Found: 153.0191

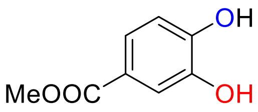

2n
methyl 3,4-dihydroxybenzoate

The title compound was obtained in 95% yield as white solid; ^{1}H NMR (500 MHz, Chloroform-*d*) δ 7.65 (d, *J* = 2.0 Hz, 1H), 7.56 (dd, *J* = 8.3, 2.0 Hz, 1H), 6.91 (d, *J* = 8.3 Hz, 1H), 6.32 (s, 0H), 6.14 (s, 0H), 3.89 (s, 2H). ^{13}C NMR (126 MHz, $CDCl_3$) δ 167.46, 148.96, 143.24, 124.06, 122.82, 116.90, 115.09, 52.25. HRMS (ESI): Calcd. for C8H8O4 (M-H): 167.0344; Found: 167.0349.

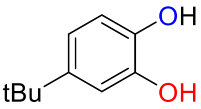

2o

4-(*tert*-butyl)benzene-1,2-diol

The title compound was obtained in 80% yield as white solid; ^{1}H NMR (400 MHz, Chloroform-*d*) δ 6.92 (d, *J* = 1.9 Hz, 1H), 6.83 – 6.78 (m, 2H), 5.39 (s, 2H), 1.27 (s, 9H). ^{13}C NMR (101 MHz, CDCl$_3$) δ 144.92, 143.18, 141.09, 117.84, 115.08, 113.12, 34.32, 31.61. HRMS (ESI): Calcd. for C10H14O2 (M-H): 165.0916; Found: 165.0922.

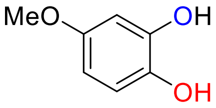

2p

4-methoxybenzene-1,2-diol

The title compound was obtained in 72%yield as white solid; ^{1}H NMR (400 MHz, Chloroform-*d*) δ 6.76 (t, *J* = 8.3 Hz, 1H), 6.60 (d, *J* = 8.3 Hz, 1H), 6.47 (d, *J* = 8.3 Hz, 1H), 5.39 (s, 2H), 3.88 (s, 3H). ^{13}C NMR (101 MHz, CDCl$_3$) δ 147.19, 144.24, 132.65, 119.95, 108.96, 103.28, 56.30. HRMS (ESI): Calcd. for C7H8O3 (M-H): 139.0395; Found: 139.0401.

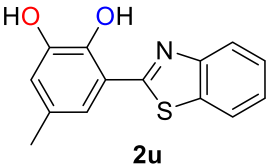

2u

3-(benzo[*d*]thiazol-2-yl)-5-methylbenzene-1,2-diol

The title compound was obtained in 76%yield as white solid; ^{1}H NMR (500 MHz, Chloroform-*d*) δ 12.58 (s, 1H), 7.98 (d, J = 8.1 Hz, 1H), 7.90 (d, J = 7.9 Hz, 1H), 7.51 (t, J = 7.6 Hz, 1H), 7.41 (t, J = 7.6 Hz, 1H), 7.03 (s, 1H), 6.88 (d, J = 1.7 Hz, 1H), 5.76 (s, 1H), 2.33 (s, 3H).^{13}C NMR (126 MHz, CDCl$_3$) δ 169.59, 151.96, 145.42, 143.39, 132.94, 129.58, 126.87, 125.68, 122.23, 121.74, 119.19, 118.44, 116.17, 20.97. HRMS (ESI): Calcd. for C14H12NO2S (M+H): 258.0589; Found: 258.0584.

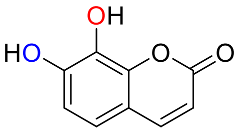

2v

7,8-dihydroxy-2*H*-chromen-2-one

The title compound was obtained in 87% yield as white solid; ^{1}H NMR (500 MHz, DMSO-*d*$_6$) δ 9.73 (s, 2H), 7.88 (d, *J* = 9.4 Hz, 1H), 7.01 (d, *J* = 8.4 Hz, 1H), 6.79 (d, *J* = 8.3 Hz, 1H), 6.17 (d, *J* = 9.3 Hz, 1H). ^{13}C NMR (126 MHz, DMSO) δ 160.31, 149.66, 144.97, 143.71, 132.09, 118.77, 112.45, 112.02, 111.15. HRMS (ESI): Calcd. for C9H7O4 (M+H): 179.0344; Found: 179.0339.

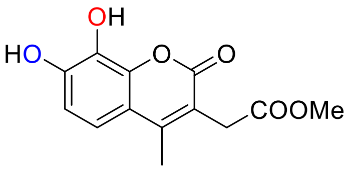

2w

methyl 2-(7,8-dihydroxy-2-oxo-2*H*-chromen-3-yl)acetate

The title compound was obtained in 81% yield as white solid; ^{1}H NMR (500 MHz, Methanol-d4) δ 7.19 (d, J = 8.7 Hz, 1H), 6.86 (d, J = 8.8 Hz, 1H), 3.74 (s, 5H), 2.41 (s, 3H). ^{13}C NMR (126 MHz, MeOD) δ 173.03, 163.63, 152.41, 150.28, 143.52, 133.33, 117.00, 116.28, 114.85, 113.50, 52.61, 33.42, 15.45. HRMS (ESI): Calcd. for C13H13O6 (M+H): 265.0712; Found: 265.0721.

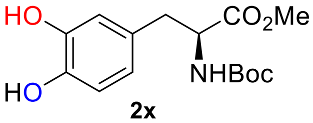

2x

Protected L-dopa

methyl (*S*)-2-((*tert*-butoxycarbonyl)amino)-3-(3,4-dihydroxyphenyl)propanoate

The title compound was obtained in 80% yield as white solid; ^{1}H NMR (500 MHz, DMSO-*d*$_6$) δ 7.12 (d, *J* = 7.9 Hz, 1H), 6.64 – 6.56 (m, 2H), 6.45 (dd, *J* = 8.0, 1.7 Hz, 1H), 4.10 – 4.00 (m, 1H), 3.59 (s, 3H), 2.78 (dd, *J* = 13.8, 5.3 Hz, 1H), 2.72 – 2.61 (m, 1H), 1.34 (s, 9H). ^{13}C NMR (126 MHz, DMSO) δ 172.64, 155.29, 144.86,

143.80, 128.13, 119.69, 116.36, 115.29, 78.19, 55.50, 51.55, 35.97, 28.07. HRMS (ESI): Calcd. for C15H21NO6 (M+Na): 334.1267; Found: 334.1265.

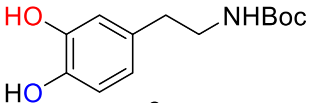

2y
Protected Dopamine
tert-butyl (3,4-dihydroxyphenethyl) carbamate

The title compound was obtained in 85% yield as white solid; ^{1}H NMR (500 MHz, DMSO-d_6) δ 8.73 (s, 1H), 8.63 (s, 1H), 6.79 (s, 1H), 6.64 (d, *J* = 7.9 Hz, 1H), 6.58 (d, *J* = 1.7 Hz, 1H), 6.44 (d, *J* = 7.9 Hz, 1H), 3.06 (d, *J* = 7.6 Hz, 2H), 2.52 (d, *J* = 8.3 Hz, 2H), 1.39 (s, 9H). ^{13}C NMR (126 MHz, DMSO) δ 155.44, 144.96, 143.41, 130.19, 119.12, 115.94, 115.42, 77.38, 41.89, 34.95, 28.22. HRMS (ESI): Calcd. for C13H19NO4 (M+Na): 276.1212; Found: 276.1215.

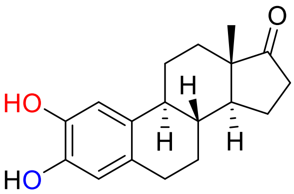

2z
Estradiol

The title compound was obtained in 72% yield as white solid; ^{1}H NMR (500 MHz, Chloroform-*d*) δ 6.81 (s, 1H), 6.61 (s, 1H), 5.42 (s, 2H), 2.78 (d, *J* = 6.5 Hz, 2H), 2.51 (dd, *J* = 18.6, 8.3 Hz, 1H), 2.29 (d, *J* = 9.2 Hz, 1H), 2.19 (s, 1H), 2.14 (dd, *J* = 19.2, 9.0 Hz, 1H), 2.05 (s, 1H), 1.95 (s, 2H), 1.61 (d, *J* = 12.3 Hz, 1H), 1.58 – 1.54 (m, 1H), 1.54 – 1.49 (m, 1H), 1.49 – 1.45 (m, 2H), 1.44 – 1.37 (m, 1H), 0.91 (s, 3H). ^{13}C NMR (126 MHz, $CDCl_3$) δ 141.87, 141.75, 132.54, 129.18, 115.71, 112.68, 50.66, 48.24, 44.23, 38.49, 36.07, 31.80, 28.92, 26.83, 26.22, 21.75, 14.04. HRMS (ESI): Calcd. for C18H22O3 (M-H): 285.1491; Found: 285.1483.

Supplementary discussion

I. Identification of catalytically active species

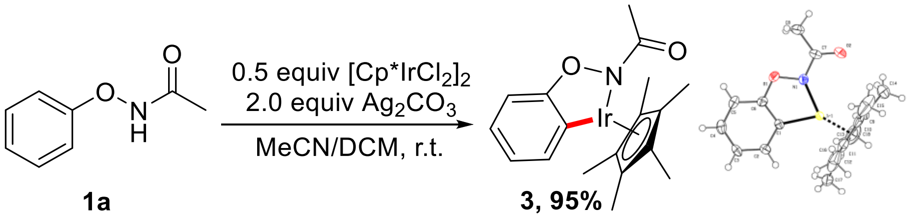

Characterization of iridium species 3:

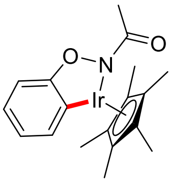

3

The title compound was obtained in 95% yield as dull-red solid; ^{1}H NMR (400 MHz, Methylene Chloride-*d*$_2$) δ 8.39 (d, J = 7.8 Hz, 1H), 7.50 (d, J = 8.2 Hz, 1H), 7.22 – 7.06 (m, 1H), 6.95 – 6.80 (m, 1H), 2.60 (d, J = 0.8 Hz, 3H), 1.94 (s, 15H). ^{13}C NMR (101 MHz, CD_2Cl2) δ 178.68, 167.59, 152.13, 137.12, 128.17, 119.12, 108.13, 92.20, 22.13, 10.68. HRMS (ESI): Calcd. for C18H22IrNO2 (M+H): 478.1358; Found: 478.1360.

Experiments on identifying catalytically active species:

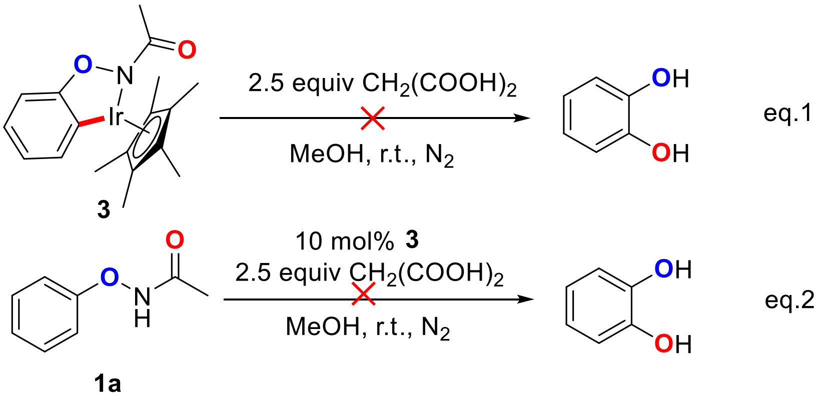

Whether species **3** was used as the catalyst nor intermediate under the standard reaction conditions led to no expected products, suggesting the five-membered iridium species **3** was not the catalytically active intermediate.

II. Theoretical calculations

a) Computational details:

All the calculations were performed with Gaussian 09 package.[5] Geometry optimization of all stationary points in gas phase was conducted by B3LYP method.[6] The LANL2DZ basis set[7] with ECP was used for Ir, and the 6-31G(d) basis set[8] was used for other atoms. Frequency analysis was performed for each optimized structure to verify the stationary points as either minima or saddle point at the same level of theory. Solvent effect (solvent=methanol) was included by single-point energy calculation using SMD[9] solvation model and B3LYP-D3 method[10] with the def2-TZVP

basis set for Ir and 6-311++G(d, p) basis set for the other atoms.[11] All relative energies (corrected with zero point energy) and Gibbs free energies (at 298.15 K and 1 atm) are reported in kcalmol^{-1}.

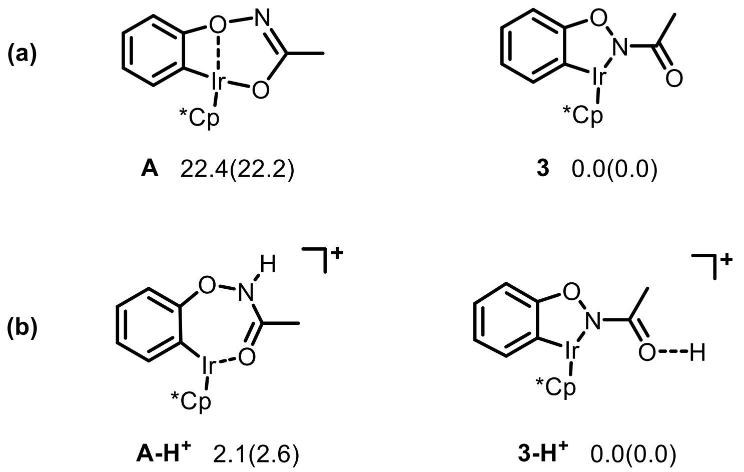

Supplementary Figure 29. Intermediate **A** and intermediate **3** under netural state **(a)** and protonated state **(b)**. Relative free energy and electronic energy (in parentheses) in solution are given in kcal/mol.

For netural mode, the free energy of species **A** is about 22.4 kcal/mol much higher than that of intermediate **3**, which indicates that in netural or basic condition intermediate **3** is favored. While by introducting acid additives, species **A** could be stablized to a large extent. The energy difference is only about 2.1 kcal/mol between protonated **A-H**$^+$ and the **3-H**$^+$ one. This two species are compatible.

b) Mass spectrometric experiments

Experimental details:

Experiments were carried out using a Synapt G2-S high definition mass spectrometer with an electrospray ion source. The instrument was held at a capillary voltage of 3.0 kV. The instrument has a quadrupole mass filter for the selection of parent ions. Mass selected ions enter into a linear ion trap cell which is filled with argon. The pressure is approximately 8×10^{-3} mbar. After collision, a reflectron time-of-flight (TOF) detector was used to determine the masses. The ion source block and nitrogen desolvation gas temperatures were set to 100 °C and 300 °C. For a given ion of interest, the elemental composition was confirmed by examination of the associated isotope envelopes in the source spectra. Collision induced dissociation was employed to analyze structural fragments.

The samples were diluted to millimolar by methanol (acid 0.37mM, $[Cp^*IrCl_2]_2$ 0.37mM), and then infused into ESI source at a flow rate of 5 μl min^{-1} through a syringe pump.

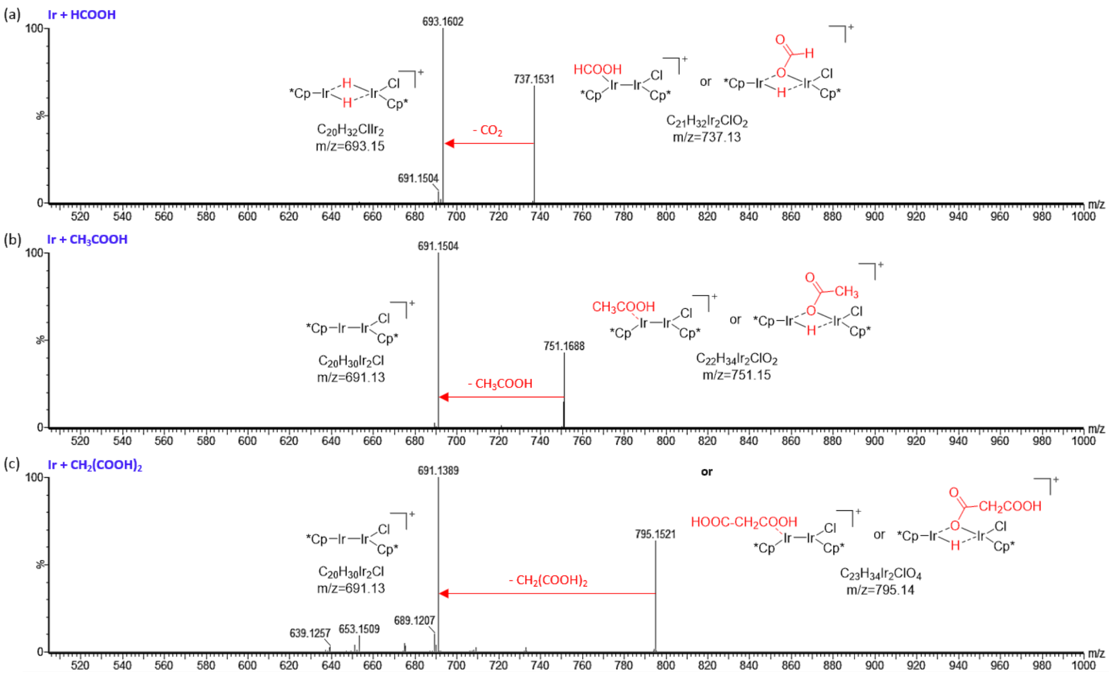

Supplementary Figure 30. (a) CID mass spectrum of ion m/z=737.15 at U_{trap}=10 V (E=0.52 eV) in 1:1 mixture of $[Cp^*IrCl_2]_2$ with HCOOH solution. **(b)** CID mass spectrum of ion m/z=751.17 at U_{trap}=12 V (E=0.61 eV) in 1:1 mixture of $[Cp^*IrCl_2]_2$ with CH_3COOH solution. **(c)** CID mass spectrum of ion m/z=795.15 at U_{trap}=17 V (E=0.81 eV) in 1:1 mixture of $[Cp^*IrCl_2]_2$ with $CH_2(COOH)_2$ solution. (Ar is used as collision gas.)

A series of methanol solutions of a 1:1 mixture of $[Cp^*IrCl_2]_2$ (0.37 mM) with HCOOH, CH_2COOH, $CH_2(COOH)_2$ acids were subjected to ESI-MS analysis respectively. Peaks m/z=737, 751 and 795 were observed in the three different samples, were preliminarily assigned to $[Cp^*{}_2Ir_2ClCHO_2]^+$, $[Cp^*{}_2Ir_2ClC_2H_4O_2]^+$, $[Cp^*{}_2Ir_2ClC_3H_4O_4]^+$. To further characterize the structure of these ions, collision induced dissociation (CID) were performed. After collision with Ar, ions m/z=751 and 795 in CH_3COOH, $CH_2(COOH)_2$ solutions dissociate one molecule CH_3COOH and $CH_2(COOH)_2$ respectively (Figure S2(b) and (c)). However, the ion m/z=737 in HCOOH system, loses a molecule CO_2 fragment (Figure S2(a)). This may give a hint why HCOOH failed for this reaction while with the addition of CH_3COOH or $CH_2(COOH)_2$ can afford the target product. The preliminary mass spectrometric result shows different reaction patterns in the presence of different acids, which may relate to the role of acid. Further comprehensive mechanistic studies are undergoing and will be covered in a following full paper.

Supplementary References

(1) Endo, Y., Shudo, K., & Okamoto, T. An acid catalyzed rearrangement of *O*-aryl-*N*-benzoylhydroxylamines; synthesis of catechols from phenols. *Synthesis*. **6,** 461-463 (1980).

(2) Li, B., Lan, J., Wu, D., & You, J. Rhodium(III)-catalyzed *ortho*-heteroarylation of phenols through Internal oxidative C-H activation: rapid screening of single-molecular white-light-emitting materials. *Angew. Chem. Int. Ed.* **127,** 14214-14218 (2015).

(3) Petrassi, H. M., Sharpless, K. B., & Kelly, J. W. The copper-mediated cross-coupling of phenylboronic acids and *N*-hydroxyphthalimide at room temperature: synthesis of aryloxyamines. *Org. Lett.* **3,** 139-142 (2001).

(4) Liu, G., Shen, Y., Zhou, Z., & Lu, X. Rhodium(III)-catalyzed redox-neutral coupling of *N*-phenoxyacetamides and alkynes with tunable selectivity. *Angew. Chem. Int. Ed.* **125,** 6149-6153 (2013).

(5) Frisch, M. J., Trucks, G. W., Schlegel, H. B., Scuseria, G. E., Robb, M. A., Cheeseman, J. R., Scalmani, G., Barone, V., Mennucci, B., Petersson, G. A., Nakatsuji, H., Caricato, M., Li, X., Hratchian, H. P., Izmaylov, A. F., Bloino, J., Zheng, G., Sonnenberg, J. L., Hada, M., Ehara, M., Toyota, K., Fukuda, R., Hasegawa, J., Ishida, M., Nakajima, T., Honda, Y., Kitao, O., Nakai, H., Vreven, T., Montgomery, J. A., Jr., Peralta, J. E., Ogliaro, F., Bearpark, M., Heyd, J. J., Brothers, E., Kudin, K. N., Staroverov, V. N., Keith, T., Kobayashi, R., Normand, J., Raghavachari, K., Rendell, A., Burant, J. C., Iyengar, S. S., Tomasi, J., Cossi, M., Rega, N., Millam, J. M., Klene, M., Knox, J. E., Cross, J. B., Bakken, V., Adamo, C., Jaramillo, J., Gomperts, R., Stratmann, R. E., Yazyev, O., Austin, A. J., Cammi, R., Pomelli, C., Ochterski, J. W., Martin, R. L., Morokuma, K., Zakrzewski, V. G., Voth, G. A., Salvador, P., Dannenberg, J. J., Dapprich, S., Daniels, A. D., Farkas, O., Foresman, J. B., Ortiz, J. V., Cioslowski, J. & Fox, D. J. *Gaussian 09, Rev. D.01*, Gaussian, Inc.: Wallingford, CT, 2010.

(6) a) Becke, A. D. Density-functional exchange-energy approximation with correct asymptotic behavior. *Phys. Rev. A: Gen. Phys.* **38**, 3098-3100 (1988);

(7) Becke, A. D. Density-functional thermochemistry. III. The role of exact exchange. *J. Chem. Phys.* **98**, 5648-5652 (1993);

(8) Lee, C., Yang, W. & Parr, R. G. Development of the Colle-Salvetti correlation-energy formula into a functional of the electron density. *Phys. Rev., B.* **37**, 785-789 (1988).

(9) a) Hay, P. J. & Wadt, W. R. Ab initio effective core potentials for molecular calculations. Potentials for K to Au including the outermost core orbitals. *J. Chem. Phys.* **82**, 299-310 (1985);

(10) Ehlers, A. W., Böhme, M., Dapprich, S., Gobbi, A., Höllwarth, A., Jonas, V., Köhler, K. F., Stegmann, R., Veldkamp, A. & Frenking, G. A set of f-polarization functions for pseudo-potential basis sets of the transition metals Sc-Cu, Y-Ag and La-Au. *Chem. Phys. Lett.* **208**, 111-114 (1993);

(11) Roy, L. E., Hay, P. J. & Martin, R. L. Revised Basis Sets for the LANL Effective Core Potentials. *J. Chem. Theory Comput.* **4**, 1029-1031 (2008).

(12) a) Ditchfield, R., Hehre, W. J. & Pople, J. A. Self‐consistent molecular‐orbital methods. IX. An extended gaussian‐type basis for molecular‐orbital studies of organic molecules. *J. Chem. Phys.* **54**, 724-728 (1971);

(13) Hariharan, P. C. & Pople, J. A. The influence of polarization functions on molecular orbital

hydrogenation energies. *Theor. Chim. Acta.* **28**, 213-222 (1973).

(14) Marenich, A. V., Cramer, C. J. & Truhlar, D. G. Universal solvation model based on solute electron density and on a continuum model of the solvent defined by the bulk dielectric constant and atomic surface tensions. *J. Phys. Chem. B* **113**, 6378-6396 (2009).

(15) Grimme, S., Antony, J., Ehrlich, S. & Krieg, H. A consistent and accurate ab initio parametrization of density functional dispersion correction (DFT-D) for the 94 elements H-Pu. *J. Chem. Phys.***132**, 154104 (2010).

(16) a) Weigend, F. & Ahlrichs, R. Balanced basis sets of split valence, triple zeta valence and quadruple zeta valence quality for H to Rn: Design and assessment of accuracy. Phys. *Chem. Chem. Phys.* **7**, 3297-3305 (2005);

(17) Krishnan, R., Binkley, J. S., Seeger, R. & Pople, J. A. Self‐consistent molecular orbital methods. XX. A basis set for correlated wave functions. *J. Chem. Phys*. **72**, 650-654 (1980);

(18) Clark, T., Chandrasekhar, J., Spitznagel, G. W. & Schleyer, P. V. R. Efficient diffuse function-augmented basis sets for anion calculations. III. The 3-21+G basis set for first-row elements, Li–F. *J. Comp. Chem.* **4**, 294-301(1983);

(19) The basis sets were obtained from the Gaussian Basis Set Library EMSL at https://bse.pnl.gov/bse/portal.
